# Rain-induced bioecological resuspension of radiocaesium in a polluted forest in Japan

**DOI:** 10.1038/s41598-020-72029-z

**Published:** 2020-09-18

**Authors:** Kazuyuki Kita, Yasuhito Igarashi, Takeshi Kinase, Naho Hayashi, Masahide Ishizuka, Kouji Adachi, Motoo Koitabashi, Tsuyoshi Thomas Sekiyama, Yuichi Onda

**Affiliations:** 1 Graduate School of Science and Engineering, Ibaraki University, 2-1-1 Bunkyo, Mito, Ibaraki 310-8512 Japan; 2 Institute for Integrated Radiation and Nuclear Science, Kyoto University, 2 Asashiro-Nishi, Kumatori, Sennan, Osaka 590-0494 Japan; 3 Meteorological Research Institute, 1-1 Nagamine, Tsukuba, Ibaraki 305-0052 Japan; 4 Faculty of Engineering and Design, Kagawa University, 2217-20 Hayashi-cho, Takamatsu, Kagawa 761-0396 Japan; 5 Forage Crop Protection Group, Division of Livestock Feeding and Management, Central Region Agricultural Research Center, National Agriculture and Food Research Organization, 768 Senbonmatsu, Nasushiobara, Tochigi 329-2793 Japan; 6 Center for Research in Isotopes and Environmental Dynamics, University of Tsukuba, 1-1-1 Tennodai, Tsukuba, Ibaraki 305-8577 Japan; 7 Present Address: Institute for Integrated Radiation and Nuclear Science, Kyoto University and College of Science, Ibaraki University, Formerly at Center for Research in Isotopes and Environmental Dynamics, University of Tsukuba, Tsukuba, Japan; 8 Present Address: Meteorological Research Institute and Formerly at College of Science, Ibaraki University, Ibaraki, Japan

**Keywords:** Ecology, Biogeochemistry, Environmental sciences, Biomarkers, Risk factors

## Abstract

It is the conventional understanding that rain removes aerosols from the atmosphere. However, the question of whether rain plays a role in releasing aerosols to the atmosphere has recently been posed by several researchers. In the present study, we show additional evidence for rain-induced aerosol emissions in a forest environment: the occurrence of radiocaesium-bearing aerosols in a Japanese forest due to rain. We carried out general radioactive aerosol observations in a typical mountainous village area within the exclusion zone in Fukushima Prefecture to determine the impacts and major drivers of the resuspension of radiocaesium originating from the nuclear accident in March 2011. We also conducted sampling according to the weather (with and without rain conditions) in a forest to clarify the sources of atmospheric radiocaesium in the polluted forest. We found that rain induces an increase in radiocaesium in the air in forests. With further investigations, we confirmed that the fungal spore sources of resuspended radiocaesium seemed to differ between rainy weather and nonrainy weather. Larger fungal particles (possibly macroconidia) are emitted during rainy conditions than during nonrainy weather, suggesting that splash generation by rain droplets is the major mechanism of the suspension of radiocaesium-bearing mould-like fungi. The present findings indicate that radiocaesium could be used as a tracer in such research fields as forest ecology, meteorology, climatology, public health and agriculture, in which fungal spores have significance.

## Introduction

We found a novel rain-related mechanism of bioecological resuspension of radiocaesium in a contaminated area in Japan. The research background is described below. It is widely known that atmospheric aerosols are removed by rain (wet removal, including in-cloud and below-cloud scavenging). However, in recent atmospheric studies, several examples of atmospheric aerosol releases supposedly related to rain have been reported^1–8^. The existence of odours known as petricor^9^ and geosmin^10^, which occur with the start of rain or with light rain, has been acknowledged for a long time, but their formation mechanism was revealed very recently^3,7,8^. In these cases, the suspension flux from the surface overwhelms the deposition flux of the aerosols in question in the near-surface air layer. The underlying mechanisms include (1) microbubbles bursting inside raindrops upon contact with the Earth’s dried porous surface^3,7^, (2) active fungal spore dispersion due to high humidity (e.g., ref.^2,4^), and (3) aerosol bursts caused by the splashing of raindrops (e.g. ref.^11^). Details of these phenomena are given in the Discussion section. Through such mechanisms, soil organics, fungal spores, bacteria and their fragments/contents (possibly formed during the rupture process^12^) can be liberated into the air. Radiocaesium (belonging to the same chemical family as potassium) can be involved in active bioecological circulation processes and can return to the atmosphere with bioaerosol release^13,14^, which is likely to be partially induced by rain.

**Figure 1:**
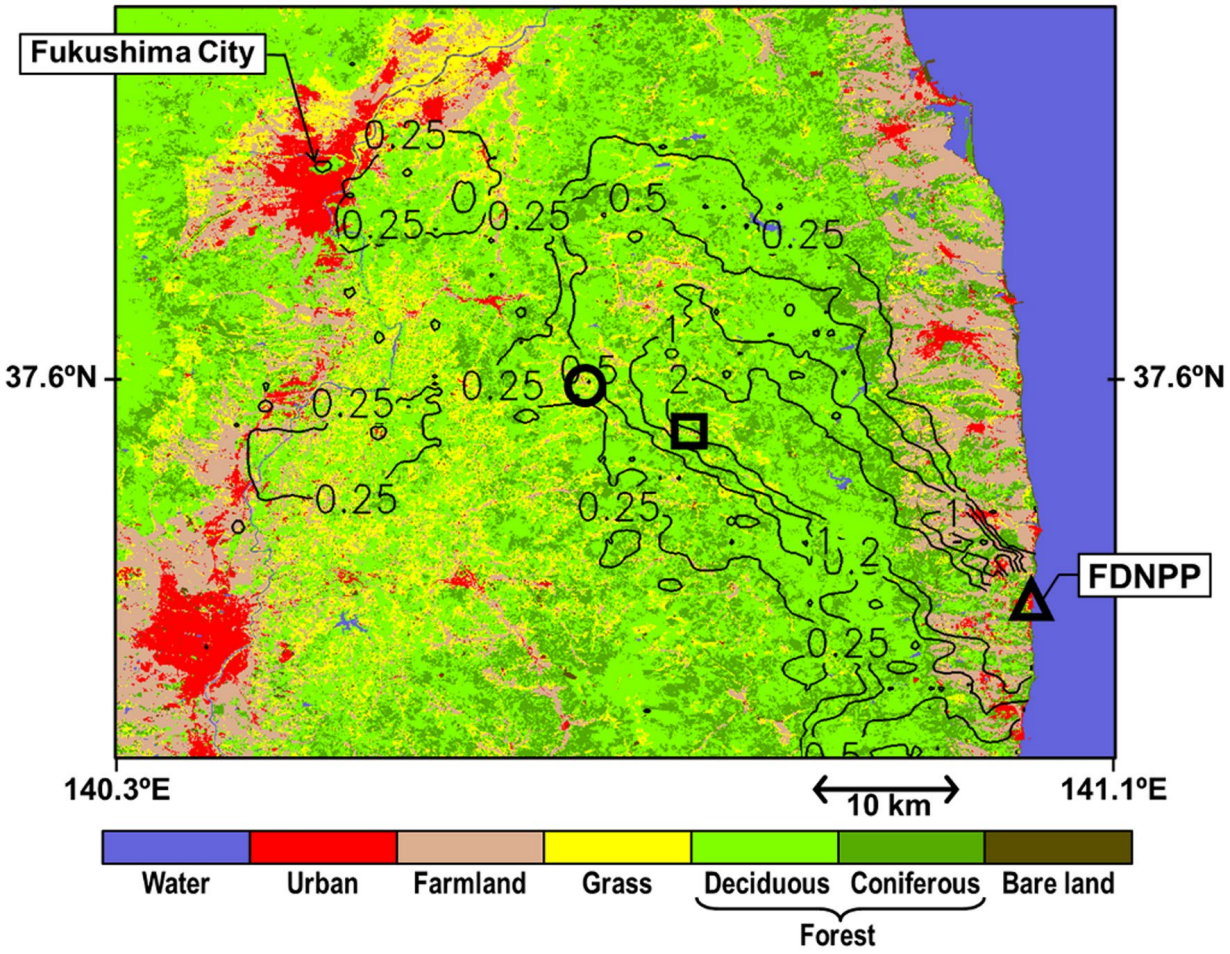
Observation site locations along with a land-cover map of the eastern part of Fukushima Prefecture before the F1NPP accident. Triangle, FDNPP (F1NPP); circle, Kawamata site; square, Namie site. The map was created by commands in GDAL version3.0.4 (open source under an X/MIT style Open Source License) and Microsoft PowerPoint 2018 for Mac and by using data from the High-Resolution Land Use and Land Cover map published by the Japan Aerospace Exploration Agency Earth Observation Research Center ALOS/ALOS-2 Science Project and the Earth Observation Priority Research: Ecosystem Research Group. The contour line shows the deposition density of ^137^Cs originating from the F1NPP accident (MBq m^−2^) at the end of May 2012^68^. Permission to use the data was granted. Deciduous forest is a mixture of various broadleaved trees, excluding evergreens. Coniferous forest excludes deciduous needleleaved trees, which are rare in the region.

We carried out atmospheric observations of radiocaesium (^134^Cs and ^137^Cs) initially originating from the Fukushima Dai-ichi Nuclear Power Plant (F1NPP or FDNPP) accident in March 2011^15^ to determine its concentrations, the processes involved in its aerosolization and the corresponding carrier^13,14,16^. Although the initial primary emission surge from the F1NPP site by the accident decreased circa the fall of 2011^17,18^, radiocaesium has been detected continuously in the atmosphere since 2011. The source of these continuous atmospheric radiocaesium levels is considered to be resuspension (i.e., secondary emissions from polluted surfaces^19^); notably, the measured radiocaesium concentrations in the range of 10^–1^ to 10^–5^ Bq m^−3^ (Supplementary Information Figure S1) have not reached a level with certain health impacts (see Annex in Igarashi et al.^13^). In a typical mountainous village area in Fukushima (see Fig. 1 and Supplementary Photographs 1 and 2), we attempted to identify the key resuspension processes and carriers of radiocaesium in the atmosphere^13,14,20^. A Chernobyl study^21^ described radioactive particle resuspension processes, such as wind uplift of the dust from contaminated surfaces, human activity and forest fires (e.g., ref.^22,23^). The Japanese summer is characterized by high rainfall and humid air, which may be unfavourable for both fugitive dust and general aerosol suspension due to wind uplift and forest fires. Furthermore, there is no evidence that photochemical reactions produce a burst of radiocaesium-bearing aerosols. We assume no emission/liberation of volatile organic Cs compounds under environmental temperatures (if any salt forms) from biota, as Cs is an alkaline metal. Our previous conclusion is that in cold seasons, a typical major driver of resuspension is the uplift of contaminated soil dust by gusts^16,20^, while in warm seasons, the major factors are bioaerosols, including contaminated fungal spores^13,14^ and cedar pollen^24^. Suspension of contaminated pollen was reported 6 years in Germany after the Chernobyl nuclear power plant accident^25^. Deposited radiocaesium was absorbed and strongly fixed by soil minerals, and a limited portion was taken up by vegetation. The time lapse from the accident suggests that the radiocaesium in pollen was related to water-soluble radiocaesium in the upper soil layer. Fungi are also a well-known bio-concentrator of radiocaesium (e.g., Ref.^26–28^). We refer to these biologically/ecologically mediated atmospheric phenomena as bioecological resuspension of radiocaesium.

The radiocaesium concentration in the atmosphere over the polluted mountainous village area investigated in this study is several times higher in summer than in winter^13,14^. Carbon with a biological origin in filter samples had a good correlation with radiocaesium concentrations, and there were sometimes close to 1 million bioaerosols per m^3^ in summer^13^. In addition, based on a combination of optical and electron microscopy, state-of-the-art DNA analysis, and radiological measurement, it was confirmed that fungal spores, one of the major components of bioaerosols^29,30^, were possibly the major host bioaerosol of radiocaesium (e.g., Ref.^31^) during summer^13^. A 3-D transport model study also revealed the significance of the secondary emission of radiocaesium from the forest during summer^20^. The seasonal trend of enhanced radiocaesium concentrations in summer has not changed significantly up to the present (Supplementary Figure S1). Here, we conducted specially designed sampling in a forest area in Fukushima Prefecture according to the weather, with the goal of determining the detailed radiocaesium emission mechanisms during the warm season. Our findings described below confirm that the polluted forest is the radiocaesium resuspension source^13, 14, 20^ and provide details on the rain-induced emission mechanism of radiocaesium-bearing aerosols during the Japanese wet summer. It is shown that rain may induce bursts of radiocaesium-bearing aerosols (coarse bioaerosols mostly of macroconidia) inside both deciduous forests and coniferous forests.

## Results

**Figure 2:**
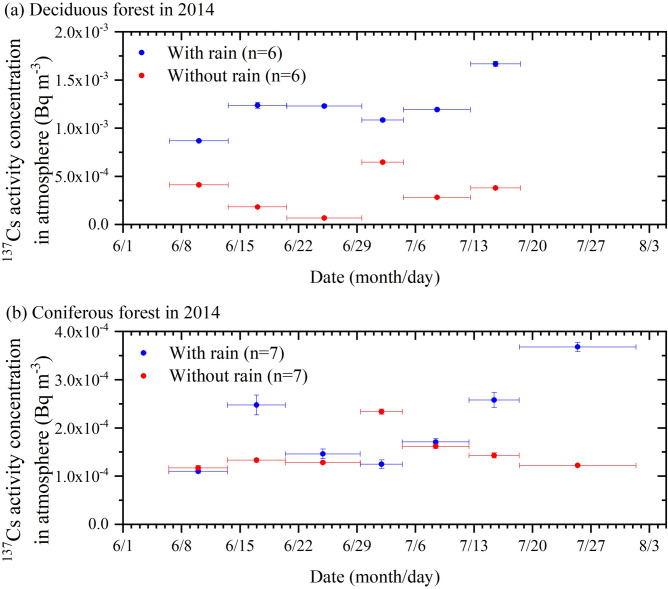
Atmospheric ^137^Cs concentration inside the contaminated forest in Fukushima Prefecture, Japan, during the summer of 2014. Rainy/nonrainy sampling was carried out from June 6 to August 2, 2014. The sampling period was shorter in the deciduous forest than in the coniferous forest. Samples collected during rain periods are shown in blue, whereas those collected during periods without rain are shown in red. Horizontal error bars indicate the whole duration of the sampling, while the vertical bars exhibit errors in the activity measurement. The top (**a**) and bottom (**b**) panels show the data from the Namie deciduous (n = 6) and Kawamata coniferous (n = 7) forests, respectively. In the deciduous forest (**a**), the ^137^Cs concentrations are always higher during the rainy period than during the nonrainy period. On the other hand, in the coniferous forest (**b**), the ^137^Cs concentrations tended to be higher during the rainy period than during the nonrainy period, except in two observation spans of June 6–13 and June 29–July 4. Caesium-137 data and sampling details are summarized in Supplementary Table S1.

In 2014 and 2016 in Fukushima Prefecture, the amount of rain was higher than usual in the rainy season (from late spring to early summer), with a few to several hundred mm of rain in each month (see Supplementary Figures S2 and S3, respectively). At the end of June 2014, a temperate cyclone (on June 29, not a typhoon) developed and brought heavy rain to northern and northeastern Japan. Additionally, in August 2016, three typhoons (Chanthu, Minduleand and Lionrock on August 16–17, 22–23 and 29–30, respectively) brought large volumes of precipitation. We conducted atmospheric sampling under both rainy and nonrainy conditions at two heavily contaminated forest sites, the Namie site and the Kawamata site (Fig. 1 and Supplementary Photographs 1 and 2), which are dominated by deciduous trees and coniferous trees, respectively. The data for 2014 are presented in Table 1 and Fig. 2. More details of the high-volume aerosol (HV) sampling results are given in Supplementary Table S1. On average, the sampling time lengths of the nonrainy periods in 2014 were approximately 2.6 times longer than those of the rainy periods in both the deciduous and coniferous forests. We found that the concentrations of ^137^Cs in the deciduous forest atmosphere with rain (average 1.21 × 10^–3^ (± 2.61 × 10^–4^) Bq m^−3^) were 2.42 times higher than those without rain (average 5.00 × 10^–4^ (± 1.89 × 10^–4^) Bq m^−3^) (Fig. 2a) on average. This difference was significant, with a p value of 0.0082 for a significance level of 1% using the paired t-test. Furthermore, this trend occurred in every consecutive sampling period. In the coniferous forests, this trend was observed in half of the sampling cases (Fig. 2b); on average, the ^137^Cs concentration during the rainy period was 1.37 times higher than that during the nonrainy period. The average difference was only significant with a p value of 0.25, giving a significance level of 25%; thus, this difference was not as clear as that in the other case. The weighted average of the radiocaesium concentration, as shown below, was also applied to the results for the deciduous forest to determine whether the difference was robust.

$$\mathop \sum \limits_{i} { }\left( {{\text{R}}_{{\text{i}}} \times {\text{ F}}_{{\text{i}}} /{\text{ F}}_{{{\text{total}}}} } \right)$$

where R_i_ is the individual ^137^Cs concentration, F_i_ is the individual sampled air volume and F_total_ represents the total sampled air volume. The ^137^Cs concentration was higher during the rainy period (1.11 × 10^–3^ (± 1.00 × 10^–4^) Bq m^−3^) than during the nonrainy period (4.68 × 10^–4^ (± 2.61 × 10^–5^) Bq m^−3^), indicating that the difference was robust.

**Table 1:** Summary of radiocaesium average concentrations in the air of the mountainous village area in the contaminated restricted zone of Fukushima Prefecture for samples with and without rain in the summer of 2014.

Sampling site	Conditions	Sample number	Total* sampling span	^134^Cs activity conc. in air**	^137^Cs activity conc. in air**
		(n)	(yyyy/mm/dd)	(Bq m^−3^)	(Bq m^−3^)
Namie (deciduous forest)	With rain	6	2014/06/06–2014/07/18	4.79 (3.47–6.27) × 10^–4^	1.21 (0.87–1.67) × 10^–3^
	Without rain	6	2014/06/06–2014/07/18	0.19 (0.10–0.26) × 10^–4^	0.50 (0.28–0.70) × 10^–3^
Kawamata (coniferous forest)	With rain	7	2014/06/06–2014/08/01	0.76 (0.38–1.18) × 10^–4^	2.04 (1.10–3.68) × 10^–4^
	Without rain	7	2014/06/06–2014/08/01	0.52 (0.39–0.84) × 10^–4^	1.48 (1.17–2.34) × 10^–4^

*Real sampling durations were dependent on the rain sensor response, and several samples were collected during the span.

**Minimum and maximum data are shown in parentheses.

Considering that, among bioaerosols, fungal spores are major ^137^Cs carriers in Fukushima forest areas^13,14^, the different results for deciduous and coniferous forests could be caused by differences in the fungal populations or fungal phyla between the two types of forests^32^. Deciduous forests may be richer in fungal activity than coniferous forests^33^. Previous authors studied litter decomposition in coniferous and deciduous forests using the litter bag method. Their results suggested that the decomposition of litter is faster in deciduous forests than in coniferous forests (*Castanopsis eyrei*) and that the species richness of fungi in deciduous forests (*Pinus massoniana*) is greater than that from coniferous forests, as indicated by the Shannon–Weaver diversity index^33^.

**Figure 3:**
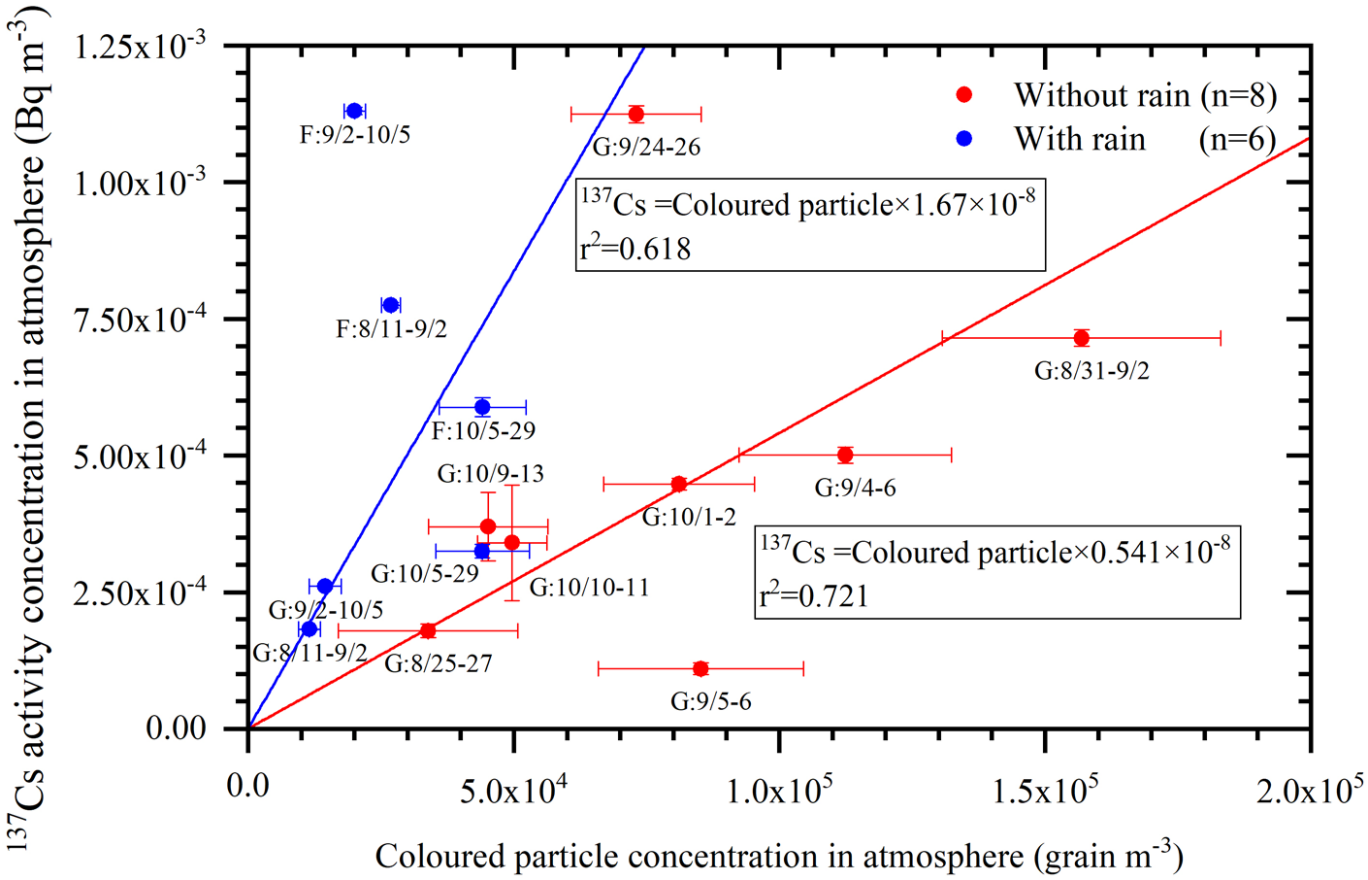
Relationship between the coloured fungal particle number concentration and ^137^Cs activity concentration in the air at the Namie site (inside the forest (F) and the bare ground (G)) during the warm season in 2016. Sampling data are expressed as mm/dd (e.g., m_1_/d_1_-m_2_/d_2_). Six of the present plotted data for the nonrainy period that had been published in Igarashi et al.^13^ were re-evaluated using the present spore counting method (see the text and Supplementary materials). The collection duration for nonrainy samples was 24 h in the daytime or nighttime (G:8/31–9/2, G:9/4–6 and G:9/24–26) of the dates shown next to each data point. For instance, daytime data of G:10/1–2 indicate that the sampling was performed from 6:00 to 18:00 on October 1 and October 2 for a total of 24 h. On the other hand, the collection duration for rainy samples encompassed several weeks due to the small percentage of the whole sampling period represented by rain. Here, regression curves were obtained by assuming that ^137^Cs was carried only by fungal spores; thus, the curves should pass the origin. Caesium-137 data and sampling details are summarized in Supplementary Table S2.

To determine the relationship between fungal particles in the air and the ^137^Cs activity concentration, we performed coloured fungal spore counting (Supplementary Figures S4 to S7) at the Namie site during the warm season in 2016; the results are shown in Fig. 3 (detailed information is given in Supplementary Table S2). Some of the data for nonrainy periods published (n = 6) in Igarashi et al.^13^ were re-evaluated using the present spore counting method. The data set (total n = 14) is a composite of those obtained at a forest site (F) and at a bare ground site (G; school ground) near the forest (Supplementary Photograph 1). Regression curves were obtained by assuming that ^137^Cs was carried only by fungal spores in order that the curves pass through the origin. Although there is uncertainty in the spore counts (see “Discussion” section), when the curves pass through the origin, fitted curves are evident, which suggests that the spore count has significance. The obtained linear relationship between the activity concentration of ^137^Cs (Y) and the fungal spore number concentration (X) in a unit volume of air during the nonrainy period is expressed as Y = 0.541 × 10^–8^ × X. The slope of the regression curve gives the ^137^Cs content in a single fungal spore, confirming the previous hypothesis that fungal spores carry radiocaesium^13^. On the other hand, the data for rainy periods exhibit the relationship of Y = 1.67 × 10^–8^ × X. Notably, the slope is approximately 3 times larger during the rainy period than during the nonrainy period.

**Figure 4:**
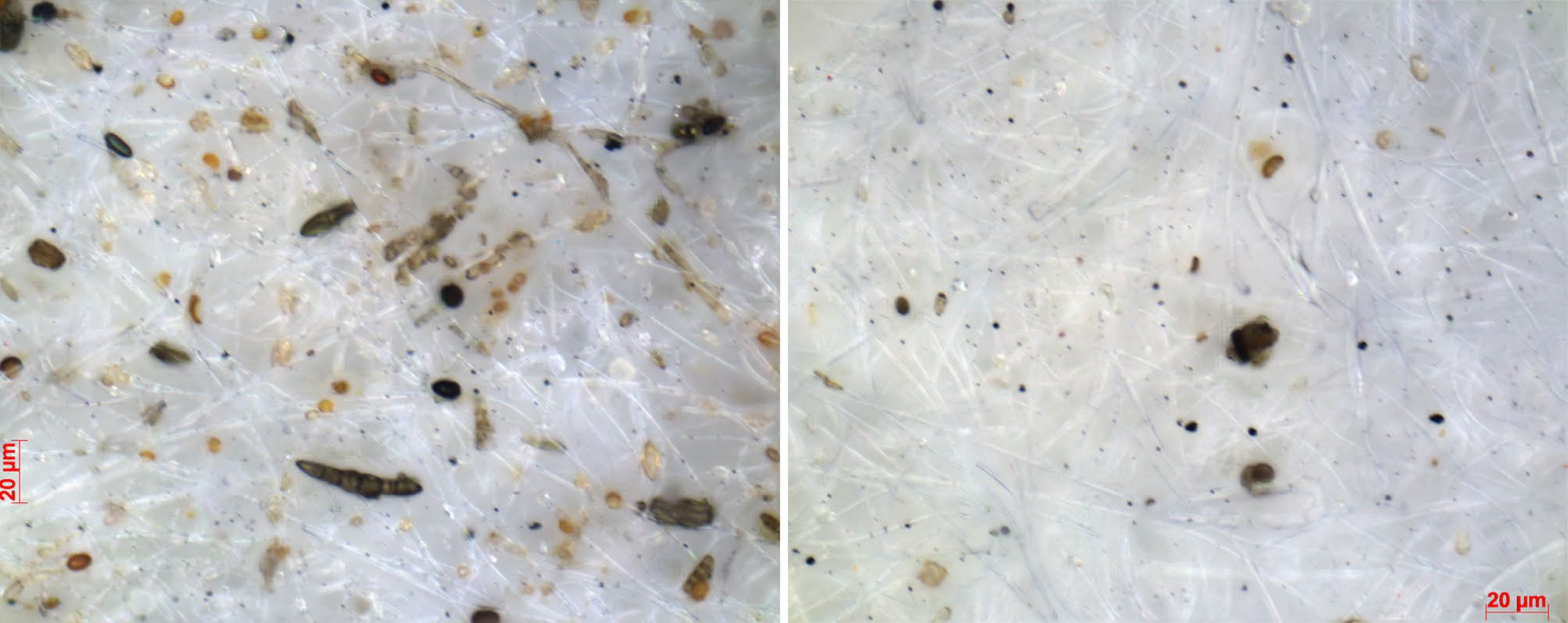
Comparison of typical optical microscopy photographs of HV filter samples (left: collected during the rainy period of September 2 to October 5, 2016, total volume of 9,094 m^3^; right: collected during the nonrainy period of September 5 to September 6, 2016, total volume of 1,296 m^3^). Samples from the rainy period display many coarse club- and oval-shaped particles, with some hypha-like materials. These are considered macroconidia. A portion of the particles exhibit sizes greater than 20 μm (red bar). On the other hand, the sample from the fine period displays many small dot-like particles of a few μm or smaller in size. Note that no size cut-off was applied during the sampling. The total pixel size of the photograph was originally 2,728 × 2,198 = 5,996,144.

This finding indicates two possibilities: (1) during the rainy period, spores with a relatively high Cs concentration are dominant or (2) the spores suspended during the rainy period have larger volumes than those suspended during the nonrainy period, although the Cs concentrations of the spores are similar during both periods. Figure 4 shows a comparison of typical optical microscopic photographs of HV filter samples collected during rainy and nonrainy periods at the bare ground site. Notably, the rainy and nonrainy collection durations differed significantly (the duration was approximately 7 times longer during rainy conditions; see the explanation of Fig. 4), resulting in differences in the particle number concentrations in the filter samples. However, Fig. 4 shows that long and coarse elliptical particles (some exceeding 20 μm), which may be macroconidia (based on size and morphology, see the Methods section), were significant components of the rainy samples.

**Figure 5:**
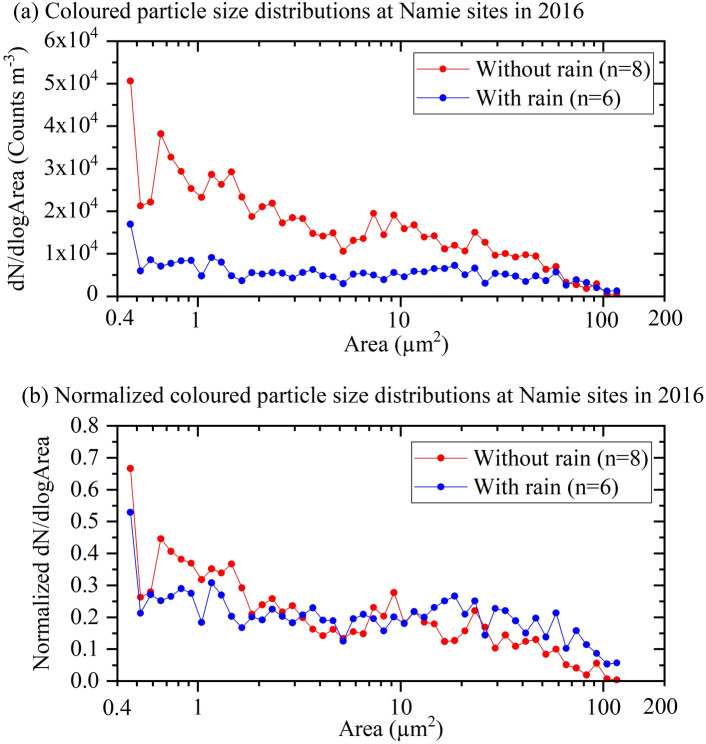
(**a**) Averaged (number concentrations per unit air volume (Y)) and (**b**) normalized (dividing by the sum of the total number (Y)) size distributions of fungal particles collected on the HV filters (n = 6 and 8 for rain and nonrain, respectively) obtained in 2016 using image analysis. In total, 4,672 and 3,764 particles were counted for nonrainy and rainy samples, respectively. The bin size of the horizontal axis (X; dlog Area) is 0.05 on the scale of the base 10 logarithm. Analysed optical microscopic images were taken from the same filter samples as those shown in Fig. 2. The size of each fungal particle is expressed in terms of the projected area. One pixel corresponds to approximately 0.008 μm^2^. Particles beyond the size of approximately 120 μm^2^ (more than 15,000 pixels) were cut-off to avoid overlapping images of particles. The scale of the typical bioaerosol sizes is the projection area shown in Supplementary Fig. 3. (**a**,**b**), respectively, reveal that the total number concentrations of coloured fungal spores decreased during rainy periods compared to during nonrainy periods (0.34) and that the portion of large spores (larger than approximately 15 µm^2^) increased from 0.19 (nonrainy period) to 0.31 (rainy period), an increase of 1.75 times.

**Figure 6:**
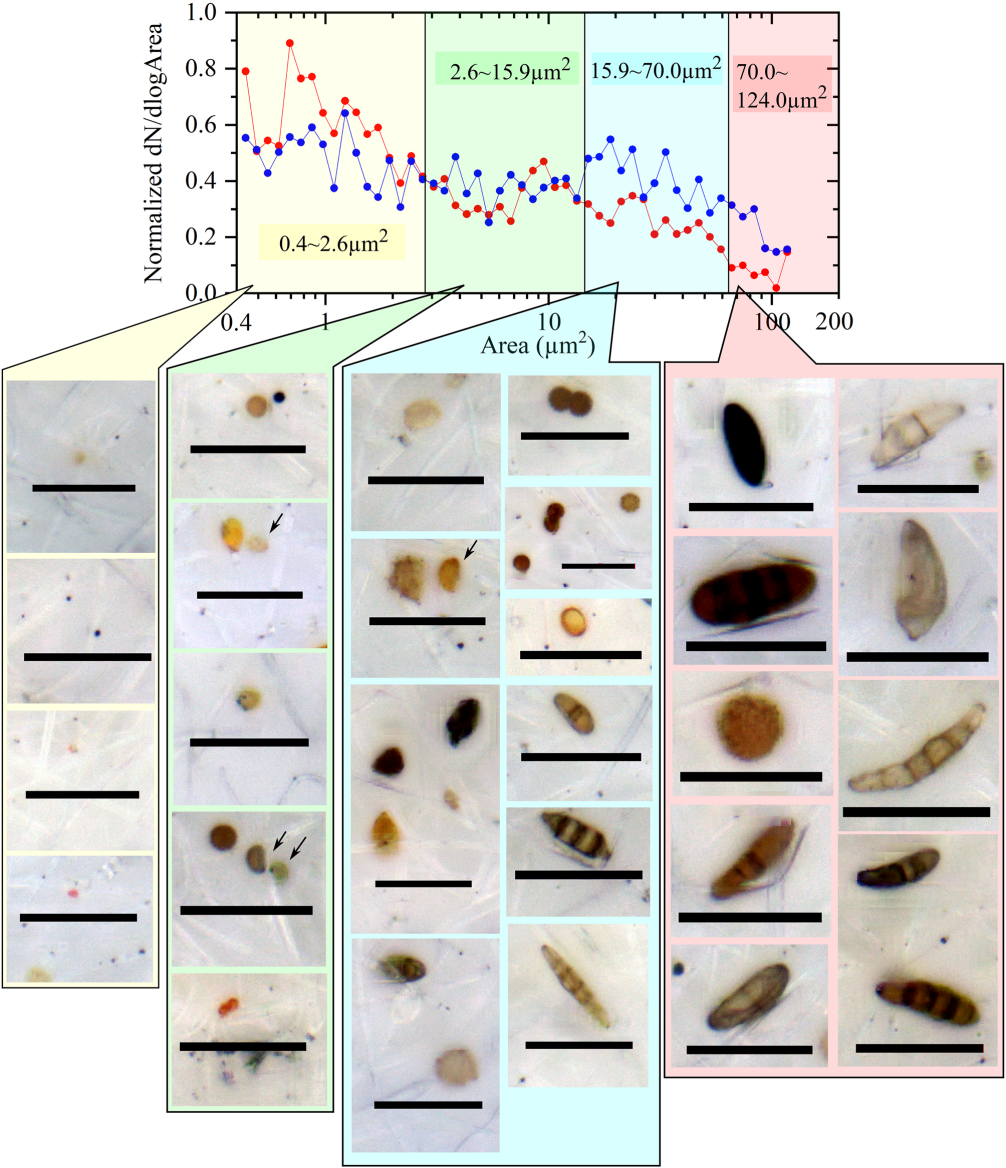
Typical examples of fungal spore particles on the HV filter samples taken at the Namie site during the 2016 summer, which are plotted along 4 projection size bins based on experimental/convenient classification. The size distribution plot is from Fig. 5b. Arrows indicate the particles concerned, and the bar length is 20 μm. Fungal spore particles are sorted according to the projection area. Beyond the 15 μm^2^ range, macroconidia were dominant, as shown in this figure.

To address the abovementioned hypothesis, we investigated whether there were changes in the size distribution of bioaerosol particles between rainy and nonrainy periods. Figure 5a shows the average size distribution of bioaerosols (projection area) for periods with and without rain, while Fig. 5b presents the normalized distribution (Supplementary Figure S8 presents the individual data). One pixel represents approximately 0.008 μm^2^ (Supplementary Figure S5 for reference). In Fig. 5a,b, the bin width W is set as follows:

$${\text{W}}\, = \,{\log}_{{{1}0}} \left( {{\text{Area}}\left( {\text{i}} \right)} \right) \, - {\log}_{{{1}0}} \left( {{\text{Area}}\left( {{\text{i}} - {1}} \right)} \right)\, = \,0.0{5}$$

where Area(i) and Area(i−1) express the ith and (i−1)th bins’ highest edges, respectively. Therefore, the summation of the normalized size distribution of dN/dlogArea is dN/(W)/$\sum \mathrm{N}$, yielding 10 instead of unity (Fig. 5b). Figure 5a indicates that the average total number of fungal spores suspended in rainy periods was significantly less than that in nonrainy periods (with a ratio of 0.34). However, Fig. 5b shows that the portion of particles larger than approximately 15 μm^2^ was higher in rainy periods than in nonrainy periods (1.75 times; proportion in nonrainy periods: 0.19, proportion in rainy periods: 0.3) and that more particles finer than approximately 3 μm^2^ were suspended in nonrainy periods than in rainy periods (1.24 times; proportion in nonrainy periods: 0.57, proportion in rainy periods: 0.46). Considering the results shown in Figs. 4, 5, 6, different types of bioaerosols (undoubtedly fungal spores) are emitted under rainy conditions than under fine weather conditions. The larger fungal spores released during rainy weather are macroconidia (often with multiple septa) according to the literature^34–37^ and based on size and morphology. Although we need more evidence to support these results (see the Discussion section), the coarse elliptical particles resemble the conidia of graminicolous fungi, such as Bipolaris, Exserohilum and Drechslera, as described in the abovementioned NARO encyclopaedia^34^. Photographs of fungal particles appearing in Fig. 6 validate our conclusion; beyond a 15 μm^2^ projection size range, macroconidia are evident.

## Conclusions

Compared to nonrainy conditions, rain induces the release of approximately twice as much radiocaesium-bearing coarse bioaerosols (especially with a projection size of > 15 μm^2^) into the atmosphere in a polluted temperate forest range in Japan, although the total number concentration of bioaerosols is reduced to approximately one-third under rainy conditions. Macroconidia particles (e.g., ref.^34^) may represent the coarse bioaerosol particles based on the analysis of size and morphology (see Fig. 6 and the Discussion section, too). Therefore, one of the mechanisms behind the summer maxima in radiocaesium over the polluted forest^13,14,20^ was revealed to be rain splash (e.g., ref.^11,38–40^). As Fukushima forests are ordinary temperate forests, the finding has many implications for forest ecology, meteorology, climate, public health, agriculture, and other fields (see the Discussion section) in which fungal spores play significant roles. However, there are limitations to the present study; we investigated the increase in bioaerosols on the basis of only radiocaesium and coloured spores, while other bioaerosol components such as organics^41,42^ were not studied in detail. In future research, sampling and measurements with increased temporal resolution (e.g., hourly) using a real-time monitoring tool, for instance, UV-APS or WIBS (e.g., ref.^43^), should be applied, and a more detailed analysis of other bioaerosol components is needed. To understand the full range of rain-induced bioaerosol emission phenomena in forest areas, we need more observational research.

## Discussion

Considering the projection sizes of the coloured fungal spores shown in Supplementary Figure S5, we determined the typical volume ratio of fungal spores suspended in the air for periods with and without rain. For example, in nonrainy periods, characteristic fungal spores exhibited a spherical size of 1,400 pixels (area size: approximately 11 μm^2^). Using the circle area equation of πr^2^, r is approximately 1.9 μm. In contrast, the typical size for spores during rainy periods was larger than 4,600 pixels (approximately 38 μm^2^, as displayed in Supplementary Figure S5). We found that the aspect ratios of these particles were 1–1.8 and 2.6–3.4 for typical nonrainy and rainy periods, respectively. We calculated the average single-particle volumes by considering the rotating body of each particle with a rotation axis along the minimum particle diameter. The single-particle volumes were 4.28 × 10^6^ µm^3^ and 11.5 × 10^6^ µm^3^ for typical nonrainy and rainy periods, respectively. The ratio between the volumes of rainy and nonrainy periods was 2.69. This number is close to the ratio between the slopes during periods with and without rain for the activity concentration of ^137^Cs (Y) and the coloured fungal spore number concentration (X) in the air (Fig. 3), i.e., 1.67/0.541≃3.1. Each slope represented the relationship between ^137^Cs air concentration (Y), the coloured fungal spore number concentration (X), the ^137^Cs volume concentration (C) and the typical fungal spore volume (V) during the rainy and nonrainy periods as follows:

$${\text{Y }} = {\text{ C}} \times {\text{V}} \times {\text{X}}.$$

Therefore, we assumed the simplest case, in which only spherical spores were suspended in nonrainy periods, only spheroid (prolate) spores were suspended in rainy periods, and C remained the same. In this case, the slope ratio represents the ratio between the fungal particle volumes of the rainy and nonrainy periods. The abovementioned approximate calculation could indicate that the assumptions are close to reality. The results in Fig. 5a show a decreasing number of fungal spores suspended in the air (the total number concentrations were lower during rainy periods than during nonrainy periods; 0.34), while Fig. 5b suggests that larger fungal spores are suspended in the air during rainy periods than during nonrainy periods (the proportion of large spores > 15 µm^2^ was higher; 1.75). We concluded, as indicated in Fig. 6, that the conidia types of fungal spore, which are larger in size but have a similar Cs concentration as typical fungal spores, become predominant in the air when it rains in Fukushima forests. Therefore, in this study, we provide evidence that increases in bioaerosol concentrations occur due to rain in Japanese forest areas. Notably, this rain-induced bioaerosol phenomenon was once thought to occur only in specific forests, such as tropical rain forests^44^, boreal forests^5^ and semidry forests^2^, and has never been considered in Japan, as the country is located in the temperate climate zone.

There are three possible major mechanisms for aerosol emissions due to rain:

1. When a raindrop touches the bare, dry surface of the Earth containing many apertures (porous in nature), the raindrop does not penetrate the earth immediately, and for a short time, the raindrop retains its shape as a small water mass. Air bubbles are generated inside this water mass from the Earth’s surface and then rise. When the tiny air bubbles rupture at the rain drop surface, very tiny droplets are ejected and result in aerosol generation^3,7^. Additionally, microbubbles burst inside a raindrop touching the Earth’s surface. The emitted aerosols can transfer material that was within/on the Earth’s surface into the atmosphere; for example, this transfer explains why the smell of soil occurs during rain^3,7^. However, dry bare surfaces are scarce in the forest areas of Fukushima Prefecture, so this mechanism is not applicable in the present case.
2. Fungi disperse spores using rain and high humidity^45–48^. Active spore dispersion could be a possible major process of bioaerosol releases in response to rain. The phyla Basidiomycota and Ascomycota in the kingdom Fungi are classified according to their different spore dispersion systems. They utilize atmospheric water in a highly dedicated structure and emit spores into the air; then, the spores become entrained due to turbulence over the Earth’s surface. This is a plausible process that provides fungal spores (especially basidiospores) to the atmosphere. In our present observation, however, we could not see this effect clearly; the total number spore concentration decreased to approximately one-third during rain, as depicted in Figs. 3 and 5.
3. It has been known for a long time (for instance, ref.^49,50^) that phytopathogens (e.g., rust fungi, which belong to Ascomycota) proliferate by rain splash^35,36,38,40^. Recently, high-speed video imaging technology has been applied, and the associated physical mechanisms have been studied^11,39,51^. Pathogen-bearing tiny droplets are dispersed by rain drop impacts on vegetation leaves. A more recent study^51^ revealed that rain drop impacts induce the formation of small air vortexes, effectively liberating dry spores from leaves into the air. These studies have revealed the role of splashing in the spread of fungal pathogenic spores (mostly mould and anamorphic Ascomycota). Moreover, rain drop impacts deliver mechanical force onto the surface materials covering the leaves, branches and trunk of trees, etc., thereby liberating any surficial materials^11,39^. In addition, canopy interception losses of rainfall^52^ may play a role in the hydrological and biogeochemical cycles in forested areas^11,39,40,53^, in which a quantity of rainwater is intercepted by the forest canopy and thus does not reach the forest floor (throughfall). This interception varies between 15 and 45% in coniferous forests^54^. Possible mechanisms are described in the literature (e.g., ref.^53,55,56^). One explanation could be that water splashes evaporate^39^, which thus could leave aerosols. We hypothesize that rain splash evaporation might add more ^137^Cs to the atmosphere. Related to this, it would be interesting to know from which vertical region of the forest the emissions mostly occur: canopy or ground. The maximum height of the canopy of the Fukushima forest is 20–25 m. However, currently, we do not have the detailed data on the height distributions of bioaerosols necessary to form a conclusion. This will be a future task to be addressed. We consider litter to be important, as described later, so emissions could mainly occur from the ground.

As concluded above, rain can induce emissions of larger fungal spores (macroconidia, often with multiple septa) carrying radiocaesium. Igarashi et al.^13^ reported that the spores and debris suspended during summer over the Fukushima typical mountainous village areas belonged to the phyla Ascomycota and Basidiomycota. They also noted that “rainwater samples exhibited larger proportions of Ascomycota, represented by the orders Capnodiales, Pleosporales, Dothidiales, Helotiales, Diaporthales, Hypocreales, and Xylariales, than did air samples”. Their findings naturally suggest that rain splash also contains spore (conidia) of these orders. Furthermore, this assumption leads to the hypothesis that conidia particles may arise from mould species covering not only living trees but also contaminated litter. Litter should be covered with more mould than the living leaves of trees. This hypothesis is the most plausible for cases in which rain drops impact contaminated forest areas.

To confirm the conidia and ascospore hypothesis, we isolated and incubated fungal strains (Supplementary Figure S9) and used DNA analysis to identify the fungi. As shown in Table 2, 45 strains of fungi (4 of which were unidentified) were isolated from the four HV filter samples collected during rain in the summer of 2016 (data from the samples are shown in Fig. 3). Six strains, including *Trametes versicolor*, were derived from Basidiomycota, while the other 39 strains (87% of isolated strains) were identified as filamentous fungi derived from Ascomycota (see Table 2). These experimental results indicated that ascospores are more dominant than basidiospores in the typical mountainous village area in Fukushima during rain. In other words, the fungal spore sources in rainy weather seem to be different from those during fine weather (though the atmosphere has high humidity).

**Table 2:** Summary of isolated and identified fungi from the HV filter samples collected during the rainy period in the summer of 2016 at the Namie site.

Sample collection	Isolated and identified fungi (order level)	
Forest site in Namie during Aug. 11 to Sep. 2	*Cladosporium sphaerospermum*	Ascomycota
	*Penicillium* sp.	Ascomycota
	*Bjerkandera adusta*	Basidiomycota
	*Talaromyces* sp.	Ascomycota
	*Thanatephorus cucumeris*	Basidiomycota
	*Cephalotheca sulfurea*	Ascomycota
	*Acremonium* sp.	Ascomycota
	*Daedalea dickinsii*	Basidiomycota
Forest site in Namie during Sep. 2 to Oct. 5	*Toxicocladosporium irritans*	Ascomycota
	*Pseudocercosporella fraxini*	Ascomycota
	*Toxicocladosporium irritans*	Ascomycota
	*Thanatephorus cucumeris*	Basidiomycota
	*Tilletiopsis* sp.	Basidiomycota
	Other	
Bare ground site in Namie during Aug. 11 to Sep. 2	*Penicillium* sp.	Ascomycota
	*Cladosporium* sp.	Ascomycota
	*Trametes versicolor*	Basidiomycota
	*Cladosporium* sp.	Ascomycota
	*Oidiodendron* sp.	Ascomycota
	*Fibulomyces mutabilis*	Basidiomycota
Bare ground site in Namie during Sep. 2 to Oct. 5	*Fusicolla* sp.	Ascomycota
	*Toxicocladosporium irritans*	Ascomycota
	*Pestalotiopsis microspore*	Ascomycota
	*Fusicolla* sp.	Ascomycota
	*Arthrinium phaeospermum*	Ascomycota
	*Xylomelasma* sp.	Ascomycota
	*Pestalotiopsis microspora*	Ascomycota
	*Talaromyces purpureogenus*	Ascomycota
	*Xylomelasma* sp.	Ascomycota
	*Fusarium merismoides*	Ascomycota
	*Valsaria insitiva*	Ascomycota
	*Sordariomycetidae* sp.	Ascomycota
	*Pestalotiopsis neglecta*	Ascomycota
	*Pestalotiopsis microspora*	Ascomycota
	*Pestalotiopsis microspora*	Ascomycota
	*Hypoxylon* sp.	Ascomycota
	*Arthrinium phaeospermum*	Ascomycota
	*Penicillium* sp.	Ascomycota
	*Xylariaceae* sp.	Ascomycota
	*Hypoxylon* sp.	Ascomycota
	*Xylomelasma* sp.	Ascomycota
	*Sordariales* sp.	Ascomycota
	Others	

The identified fungi are attributed to the phyla Ascomycota or Basidiomycota. The 4 unidentified strains are expressed as other/others.

However, we do not have clear evidence that mould (Ascomycota) in general bioconcentrates radiocaesium, which mushroom fungi (mostly Basidiomycota) are known to do^26–28^. Another major uncertainty of the present study is related to the use of optical microscopy for fungal spore counting. In the present case, we counted only coloured spores (on the order of 10^4^ to 10^5^ grains per m^3^), although we tried to count faintly coloured spores as often as possible. As mentioned in the Methods section, the spore counting method itself involves errors of approximately 10%. However, the present spore counting method gives an average that is approximately 3 times higher than the average of the previous counting method of Igarashi et al.^13^. As described in a previous report^13^, “the total fungal spore number concentration, including both coloured and colourless ones, might be about one order of magnitude larger” (10^5^ to 10^6^ grains per m^3^), as shown in Fig. 3 in the report^13^. Optical microscopy with fluorescent staining may miss dark-coloured spores, while coloured spore counting disregards transparent spores. Presently, fungal spore counting is methodology dependent, which is clearly a major source of uncertainties and limitations. However, these uncertainties and limitations do not subtract from the conclusions that fungal spores are carriers of radiocaesium and that rain induces the emission of bioaerosols. Certainly, more quantitative evaluations are necessary, and therefore, the application of sequential automated bioaerosol counting, such as UV-APS^57^ or WIBS^58^, to reveal if any correlation exists among bioaerosol counts, radiocaesium and weather parameters is another attractive challenge.

We add that the number of pollen particles suspended in the air was not significant during summer, as reported in ref.^13,14^. Pollen particles can contain a considerable amount of ^137^Cs^24^; if significant numbers of these particles had been mixed with the other bioaerosols, the concentration of ^137^Cs would have increased. As explained in Igarashi et al.^13^, the major bioaerosols serving as radiocaesium carriers in summer are fungal spores, not pollen. Kinase et al.^14^ manually counted the relative numbers of “pollen” and “bacteria” (note that the latter included “spores”), representing typical bioaerosols in the warm season, using scanning electron microscopy (SEM) images and concluded that the “pollen” concentration was indeed smaller than 1/10 of the “bacteria” concentration.

Thus, one of the possible mechanisms of radiocaesium resuspension from the polluted forest environment during the wet and warm periods was revealed in this study. In other words, radiocaesium can be used as a tracer to reveal unknown processes related to bioaerosol emissions from forest environments. Although the atmospheric radiocaesium activity concentration is decreasing (Supplementary Figure S1), radiocaesium can be measured more easily and precisely than bioaerosols, as described here. We estimated the apparent half-life of ^137^Cs in air at the Namie site, and we found that at least 19 years will be necessary until the ^137^Cs concentration decreases below the limit of detection. Radiocaesium will certainly disappear in the future in the study region, and the current radiocaesium concentration level (10^–3^ to 10^–5^ Bqm^–3^) in air can indeed help us to clarify the radiocaesium resuspension process, in which bioaerosols are certainly involved. However, to model rain-induced bioaerosol emissions, further research is necessary. In addition, we disregarded the possibility of bacterial suspension into the air by rain splash^59^, although bacteria might also carry radiocaesium. Thus, to further reveal the radiocaesium cycle within the contaminated forest environment, we need to conduct additional research. In particular, we need more sophisticated definitions and measurement methodologies not only for bioaerosol counting but also emission/deposition flux observations.

No previous studies have reported the resuspension of radiocaesium by bioaerosols, namely, fungal spores, during summer in a forest, except for studies in which the present authors were involved^13,14,20^. We searched for any prior similar bioaerosol/primary biological aerosol particle (PBAP) study in Japan, but no studies have addressed rain and its relevance to the PBAP number concentration. Furthermore, even though a study on secondary organic aerosol (SOA) generation from isoprene and terpene derived from vegetation was carried out^60–62^, primary material outflow from forest ecosystems has received almost no attention. Because two-thirds of the country is covered by forest, we strongly feel that there is a need for a full-scale study on bioaerosol and/or organic matter emissions in response to rain in Japan, and the results may be applicable for all temperate mixed forests worldwide. Additionally, the emitted fungal spores released during rain are primarily mould spores, so an allergy pandemic (e.g., ref.^63^) and agricultural pathogen epidemic (e.g., ref.^11,38,39,51^) in the rainy season might occur.

It has been discussed whether fungal spores can influence the weather or climate (e.g., ref.^64^), which is also an underlying motivation of the present research. We are collecting fungal fruits and obtaining spores not only from Fukushima but also from Tsukuba, Ibaraki and are trying to analyse their ice nuclei (IN) activity. Although the results are very preliminary, an example of a basidiospore is presented in Supplementary Figure S10. The ice nucleation onset was − 18 °C for the present case. Atmospheric IN in a pine forest (Colorado, United States) were measured in the summer of 2011^2,4,64^, and the results revealed that bioaerosol and IN concentrations increased during and after rain events. These studies also found that typical IN were basidiospores^4,64^, although the bioaerosols released due to rain/high humidity varied. Huffman et al.^2^ thus noted the possibility that ascospore are also potential IN. The rain-induced spore species were different from the ones in the current study, a possible result of differences in the ecosystems or the effects of the particle size cut-off of the sampling methods. We applied no size cut-off in the HV filter sampling, which might have resulted in the observation of coarser bioaerosols in this study than in other studies. Very recently, in 2019, Iwata et al.^65^ published a study stating that rain enhances the IN number (working > − 22 °C) in the air and that some of the IN seemed to be fungal spores based on observations on the coast of the Sea of Japan. They applied an impactor with a 50% cut-off diameter of 1.1 µm for sample collection^65^; thus, they might have observed different types of bioaerosols than we did. However, the report is agreement that fungal spores, compared to other IN materials, function at high temperatures of a few degrees below 0 °C to − 15 °C (e.g., ref.^66^). Fungal spores might also work as especially large cloud condensation nuclei (CCN), referred to as a giant CCN (GCCN)^46^. GCCN can form large droplets within a shorter period of time than small CCN, thereby removing water from the air column efficiently and contributing to enhanced precipitation strength. Macroconidia have a larger size than other PBAPs and might thus work more efficiently as GCCN than other PBAPs. However, we need to confirm these hypotheses in the future.

## Methods

We have used two forest sites in a mountainous village area in the range of the evacuated zone (the administrative border is not shown) in Fukushima Prefecture: one is in Namie town and the other is in Kawamata town, as depicted in Fig. 1. This figure was created by using data from the High-Resolution Land Use and Land Cover map (JAXA EORC^67^), and the ^137^Cs contour line was drawn based on the data of Torii et al.^68^ The sampling points are also described in detail elsewhere^13,14,16,20^. The environment of the sampling sites is displayed in Supplementary Photographs 1 and 2 for reference. The Namie site is located approximately 30 km northwest of the F1NPP, and deciduous trees are dominant, although some red pine trees are present. This site is on a small hill, and the school athletic grounds (bare soil originally, though gradually covered by glasses with a few small pine trees) was within a few tens of metres. Decontamination work was later carried out within a range of 1 km (see ref.^﻿14﻿^), although most of the forest remained contaminated. The Kawamata site is approximately 6 km northwest of the Namie site, and the level of radioactive contamination is lower than that of the Namie site because the contamination by the radioactive plumes in 2011 was relatively lower. This site was an artificial conifer plantation (cedar forest) on a small hill. The contamination level of ^134^Cs and ^137^Cs was at approximately a few MBq m^-2^ at both sites in 2012, as evidenced by the contour in Fig. 1.

High-volume aerosol samplers (HV; Sibata HV 1000F and R, Tokyo, Japan) were employed to collect the resuspended ^137^Cs with carrier aerosols (see Supplementary Photographs). No size cut-off was used for the sampling. One of the two HV samplers was automated to work for an hour after a sensor (Climatec, Tokyo, Japan) detected rain, while the other HV worked when the HV sampler for rain was not in operation. The automatic switch equipment was composed of a rain sensor (CPR-PPS-03), a logger (C-CR800-4 M), a 2-channel relay control driver (C-CPC-2), an alternating current (AC) relay, a power supply, a lightning arrester (C-PT10), USB-RS232C conversion cables, etc. When the sensor detected rain drops larger than 0.5 mmϕ, HV sampling started, which continued for one hour. Therefore, we could compare ^137^Cs concentrations between periods with and without rain. The filters were made of silica fibre (Advantech QR100 or Pal flex 2,500 QAT-UP; 203 mm × 254 mm), which were treated in a furnace at 400 °C before use. The sampling was performed approximately 1.5 m above the ground from June 6 to August 1, 2014 (see Table 1 and Supplementary Table S1) at the Kawamata (rainy plus nonrainy samples, n = 14) and Namie (same as above, n = 12) sites (Supplementary Photographs 1 and 2). In the summer of 2016, a sampling campaign was conducted (same as above, n = 14) only at Namie from August 11 to October 29 (see Supplementary Table S2). After sampling, the filters were wrapped by aluminium foil and then packed in a plastic sealing bag at the site and taken back to the laboratory. At the laboratory, they were kept at room temperature mostly with desiccation in a sealed plastic case, and a portion of approximately 2% of the filter area was punched out as circles (usually 8 pieces in total 16%) and used for the bioaerosol (fungal spore) counting (2 pieces kept at room temp.) and future chemical analysis (6 pieces kept in a refrigerator). Some of the latter punched-out samples were subjected to DNA analysis. The rest of the HV filters (84%) were subjected to radioactivity measurements.

The activity of ^137^Cs in the HV filter samples was obtained by γ-ray spectrometry with an intrinsic germanium semiconductor detector (coaxial type from Ortec EG&G, Eurisys or Canberra, all from Tokyo, Japan) coupled with a computed multichannel analyser (Oxford-Tennelec Multiport or Seiko EG&G MCA7600, both from Tokyo, Japan). The detection limits of the measurement of ^134^Cs and ^137^Cs at the Meteorological Research Institute (MRI) were approximately 9 and 10 mBq per sample, respectively, for approximately 10^5^ s. The temporal change in ^137^Cs air concentrations derived from the F1NPP accident at the Namie site is shown in Supplementary Figure S1.

The fungal spore counts were performed using optical microscopy (OM). The OM instrument was an Axio Imager M2m (Carl Zeiss, Tokyo, Japan), and photographs were captured at 50 times magnification in reflection mode. A portion of the HV filter samples was placed directly on a slide glass and subjected to OM observation. The OM photograph was taken by a CCD camera (6 M pixels, Zeiss Axiocam 506 colour) equipped with self-adjustment functions for white balance and exposure time. Five sections of the OM photograph that minimized overlap and maximized the number of spore images were chosen, avoiding lumpy surfaces and pollen. To count coloured fungal spores digitally to the best extent possible, we defined a coloured particle as a particle darker than the filter fibre or as a particle with a different colour than the filter fibre. For these reasons, the original photograph was digitized by adjusting (a) contrast and brightness and (b) chroma saturation, and then coloured particles were selected. During the image retouching process with the free software ImageJ^69^, the “Brightness/Contrast” and “Color Threshold” functions were used. The “Brightness” and “Contrast” setting were adjusted during the “Brightness/Contrast” process, and “Saturation” on the “Colour Threshold” palette was adjusted to obtain clear and distinct images. The obtained images were converted into binary images, and low levels of noise were removed using the median filter for two pixels. Two binary images were combined, and a final binary image (edge detected) of coloured fungal spores was obtained. In this procedure, the overlapped image was manually separated into single particles. Additionally, the particle hang on the frame was removed. The “Analyse particle” command was finally applied, and this automated counting procedure provided statistics on the coloured fungal spores. An example of the image analysis procedure is given in Supplementary Figure S4. For spore detection, the minimum spore size was set to approximately 0.4 µm^2^ (50 pixels), and the maximum spore size was set to approximately 124 µm^2^ (15,000 pixels). This corresponds to an equivalent diameter range of 0.73–12.6 µm. A typical size analysis of the coloured bioaerosol is shown in Supplementary Figure S5. The current counting method resulted in more coloured fungal spore counts than the previous method of Igarashi et al.^13^. The differences in the particle counts are shown in Supplementary Figure S6. In the figure, the present counting method yielded 1.8 times more coloured spores than the previous method^13^, while on average, approximately 3 times more particles were identified. The present method identified a higher number of faintly coloured and small fungal spores. This is a methodological limitation, which should be solved in future studies.

Figure 5 was thus created based on the counting mentioned above. For the data plot, 584 ± 284 (n = 8; 1 s.d.) and 627 ± 316 (n = 6) particles on average were counted for nonrainy and rainy samples collected in 2016, respectively. Converting these values into totals yields 4,672 and 3,764 particles for nonrainy and rainy samples, respectively, which seem statistically significant. Additionally, the error in the spore counting was estimated on the basis of 3 factors: (1) the reproducibility of the counting and (2) and (3) the size measurement. (1) The same optical photograph (sample number NHVR-281029 Photo#6) was analysed 10 times for total spore counts, and the resulting average and standard deviation were 89.9 ± 11.1 fungal particles (relative error = 12.3%), so the fungal spore counting involves an error of approximately 10%. (2) A given scale printed on the photograph (20 µm) was measured 10 times and the average and standard deviation were obtained (average = 220.1 ± 0.43 pixels (relative error = 0.19%)). (3) Three coarse particles were measured for size 10 times (see also Supplementary Figure S7). Two spores with lengths of 13.7 and 17.4 µm yielded areas of 5,515 ± 218 pixels (relative error = 3.9%) and 9,581 ± 230 pixels (relative error = 2.4%), respectively. The largest spore was out of the current measurement range, which certainly represents a limitation of the present counting method. In total, simple summation of the errors in scale measurement and replication yields an error of less than 10%, so fungal spore counting is expected to involve an error of approximately 10%. The data reveal the current limitations of the methodology employed.

The samples subjected to DNA analysis were collected by HV filtering during rain in August and September 2016 in the deciduous forest and over bare ground (Namie). A piece of the quartz fibre filter was subjected to culturing on threefold diluted Gellan gum powder (2%, wt/vol) (plant tissue grade; Wako, Osaka, Japan) at 28 °C for a week, and a single colony was picked for further incubation. Supplementary Figure S9 displays examples of the incubated samples, indicating that they were well-isolated single species. Genomic DNA was extracted from the individual incubated samples according to the method described by Lee and Taylor^70^. Polymerase chain reaction (PCR) was performed as described by White et al.^71^ using primers for internal transcribed spacers (ITSs; ITS1 and ITS4) with *Taq* DNA polymerase (Takara Bio Inc., Kusatsu, Shiga, Japan). The PCR products were purified and then sequenced using an Applied Biosystems 3730xl DNA Analyzer (Applied Biosystems, Foster City, CA, USA). Sequencing reactions were performed employing ABI PRISM Big Dye Terminator, v 3.1 (Applied Biosystems) using the primer ITS1. Sequence data of the ITS regions were downloaded from the DNA Data Bank of Japan and the European Molecular Biology Laboratory/genomic data bank (DDBJ/EMBL/GenBank) databases, and the Basic Local Alignment Search Tool (BLAST) was utilized to search for regions of similarity between biological sequences.

Meteorological conditions can influence bioaerosol species, some of which carry ^137^Cs, and the number concentrations of bioaerosols. Therefore, precipitation data in 2014 (Supplementary Figure S2) were obtained from the AMeDAS (Automated Meteorological Data Acquisition System) Japan Meteorological Agency weather station at Tsushima (37°33.6´ N, 140°45.2´ E, altitude 400 m), which is approximately 6.2 and 1.2 km from the Kawamata and Namie sites, respectively. Additionally, we obtained data from an automated weather station (AWS) at the Namie bare ground site in 2016; these data are summarized in Supplementary Figure S3. The main types of measurements of the AWS are as follows: precipitation (Takeda Keiki Kougyou, TKF-1), wind speed (three-cup anemometer, R. M. Young, Model 3,102, and sonic anemometer, R. M. Young, Model 81,000), air temperature, and humidity (Vaisala Corp., HMP155D), with data recorded by a data logger (Campbell Scientific Inc., CR1000-4 M). Details are also given elsewhere^13,14,16^.

## Supplementary information

Supplementary information.

## Data Availability

The data that support the findings of this study are available upon request. Please contact the corresponding authors.
