# Supplementary material for: Rain-induced bioecological resuspension of radiocaesium in a polluted forest in Japan
Source: Sci Rep. 2020 Sep 18;10:15330. doi: 10.1038/s41598-020-72029-z (PMC7501248; doi:10.1038/s41598-020-72029-z)
Supplement: Supplementary file 1 — Supplementary information. [file 41598_2020_72029_MOESM1_ESM.docx]

# Supplementary Information for Rain-induced bioecological resuspension of radiocaesium in a polluted forest in Japan

Kazuyuki Kita^1^, Yasuhito Igarashi ^1,2*^, Takeshi Kinase^3**^, Naho Hayashi^1^, Masahide Ishizuka^4^,

Kouji Adachi^3^, Motoo Koitabashi^5^, Tsuyoshi Thomas Sekiyama^3^ and Yuichi Onda^6^

^1^ College of Science, Ibaraki University, 2-1-1 Bunkyo, Mito, Ibaraki, 310-8512 Japan

^2^ Institute for Integrated Radiation and Nuclear Science, Kyoto University, 2 Asashiro-Nishi, Kumatori, Sennan, Osaka, 590-0494 Japan

^3^ Meteorological Research Institute, 1-1 Nagamine, Tsukuba, Ibaraki, 305-0052 Japan

^4^ Faculty of Engineering and Design, Kagawa University, 2217-20 Hayashi-cho, Takamatsu, Kagawa, 761-0396 Japan

^5^ Forage Crop Protection Group, Division of Livestock Feeding and Management, Central Region Agricultural Research Center, National Agriculture and Food Research Organization, 768 Senbonmatsu, Nasushiobara, Tochigi 329-2793, Japan

^6^ Center for Research in Isotopes and Environmental Dynamics, University of Tsukuba, 1-1-1 Tennodai, Tsukuba, Ibaraki, 305-8577 Japan

*Presently at Institute for Integrated Radiation and Nuclear Science, Kyoto University and College of Science, Ibaraki University, formerly at Center for Research in Isotopes and Environmental Dynamics, University of Tsukuba

**Presently at Meteorological Research Institute and formerly at College of Science, Ibaraki University

**This file contains:**

Supplementary Photographs 1 to 2

Supplementary Figures S1 to S10

Supplementary Tables S1 to S2


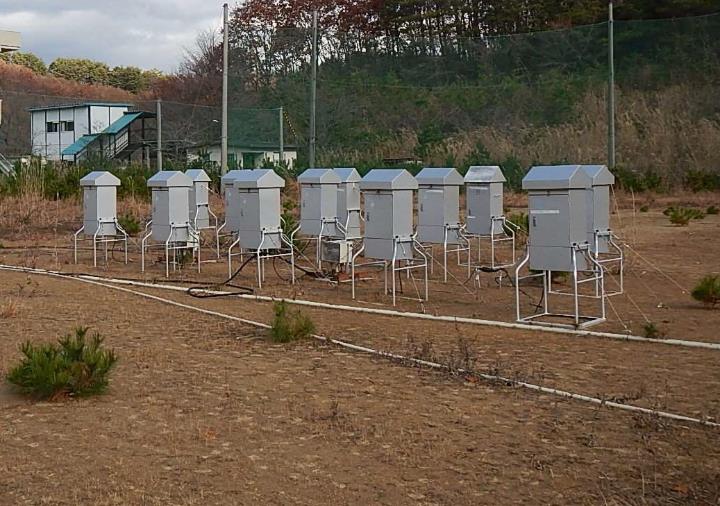


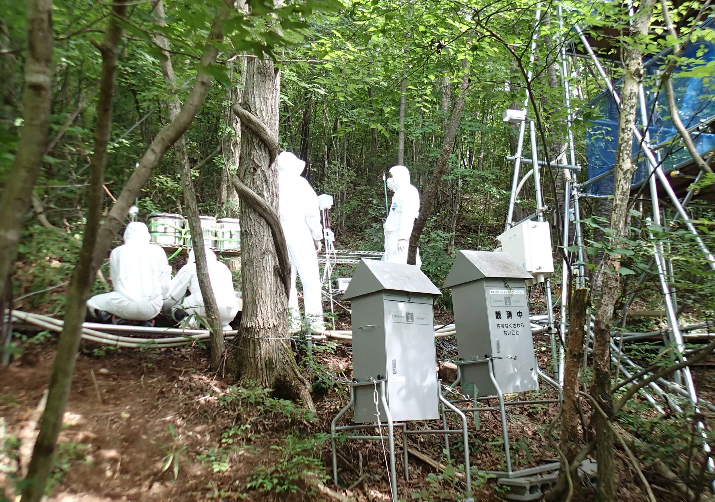


**Supplementary Photograph 1** The Namie (deciduous forest) sampling points (upper panel: school ground (G; bare soil); lower panel: forest (F)). The top photograph was taken in November 2016, and the bottom photograph was taken in August 2015. At both points, multiple HV samplers were installed and employed for the aerosol samplings. Since the evacuation of the residents in 2011, there has been no human activity at these sites, which allowed the gradual growth and development of weeds, short grasses and tiny pine trees on the school grounds (upper panel).


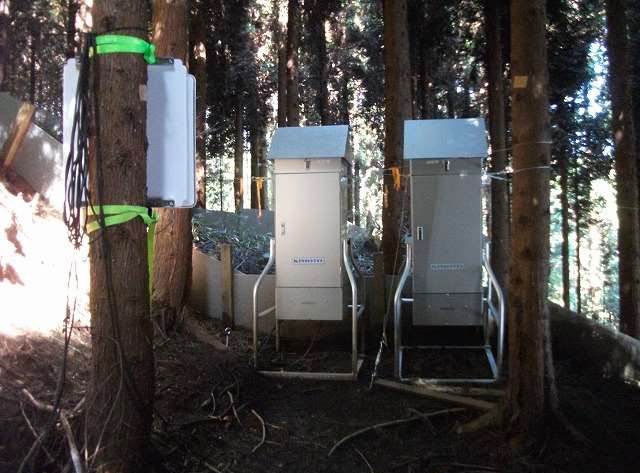


**Supplementary Photograph 2** The Kawamata (coniferous forest) sampling site taken in October 2013. Since the evacuation of the residents in 2011, there has been no human activity at the site, which has experienced little change.


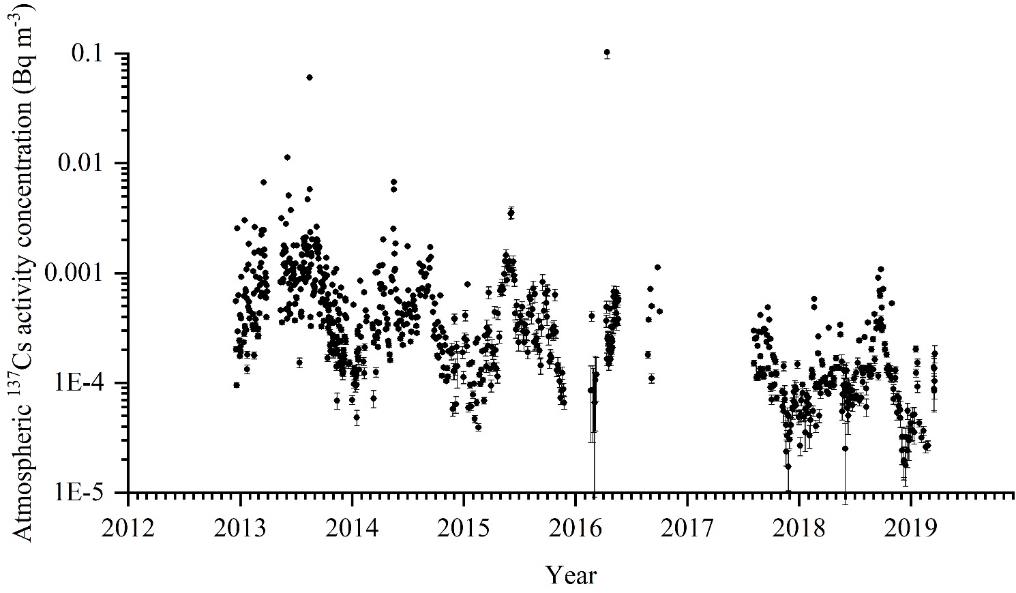


**Supplementary Figure S1** Temporal changes in ^137^Cs derived from the Fukushima Daiichi Nuclear Power Plant accident in the air over Namie, Fukushima, from 2012 to early 2019. Concentration maxima were recorded during warm seasons throughout the observation period. Observation gaps were mostly due to administrative reasons. The data prior to 2015 were published in Igarashi et al., 2019^13^.

**Supplementary Figure S2** Precipitation record (mm h^-1^) from June to July 2014 at the AMeDAS site (Tsushima), which is run by the Japan Meteorological Agency and is the nearest meteorological site to our observation site. This AMeDAS site is located approximately 6.2 and 1.2 km from the Kawamata and Namie sites, respectively. The data are given as mm/dd. From June 6 to August 2, the weather-dependent observation span, there was a total of 449.5 mm of accumulated precipitation. The minimum precipitation rate was 0.5 mm h^-1^. There was 1.7 times more precipitation in the daytime than at nighttime.


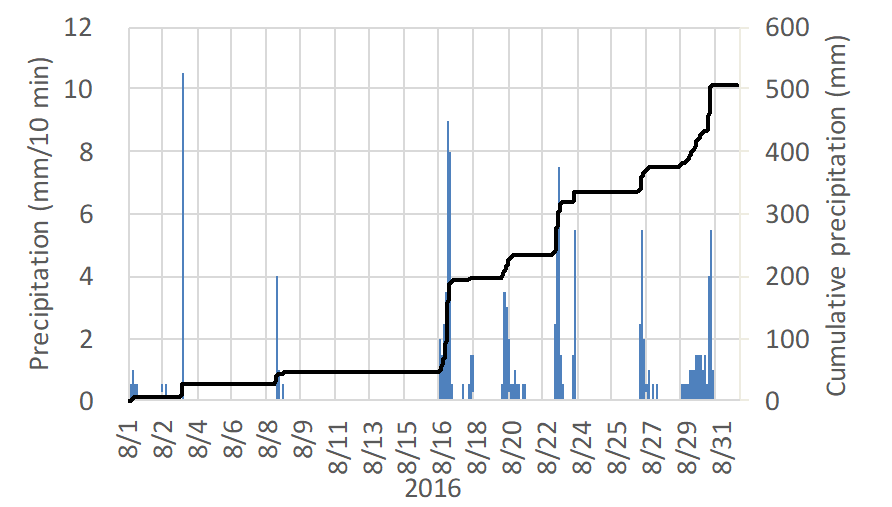


(a)


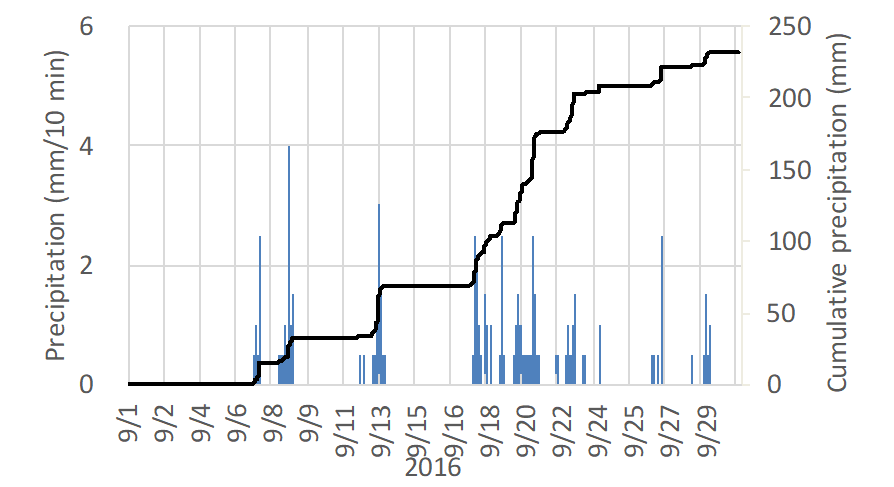


(b)

**Supplementary Figure S3** Precipitation (mm 10 min^-1^) recorded during August (a) and September (b) 2016 by an automated weather station at the Namie sampling site. The data are expressed as mm/dd in 2016.


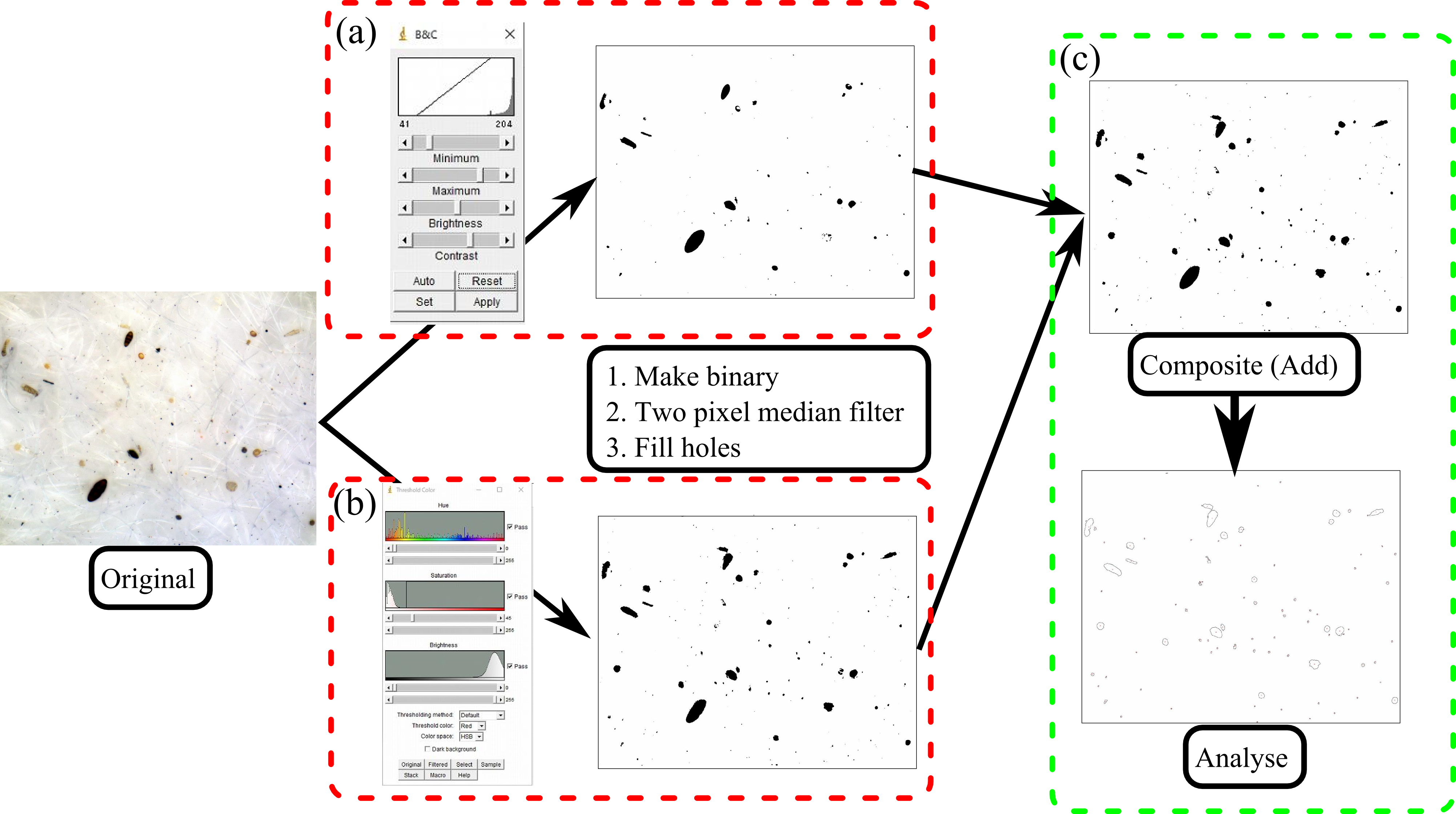


**Supplementary Figure S4** Steps in the analysis of optical microscopy photos of HV filter samples for the counting of coloured fungal spores by using ImageJ software: (a) adjust the "Brightness" and "Contrast" to find thick, dark-coloured particles; (b) adjust the "Saturation" to find faintly coloured particles; and (c) derive the final binary images synthesized from (a) and (b) of the coloured fungal spores (filled (upper) and unfilled (lower) images).


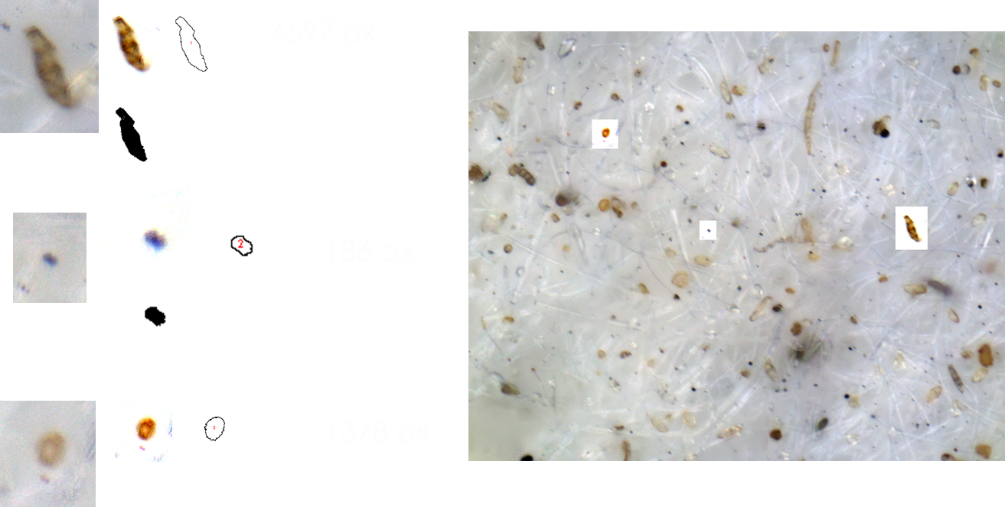


20 μm

1378 pixels

186 pixels

4597 pixels

**Supplementary Figure S5** Typical projection size analysis of the coloured bioaerosols using ImageJ image analysis software showing the binarization method and final pixel sizes. Small and large spherical spores have projection sizes of approximately 200 and 1400 pixels (approximately 1.6 and 11 μm^2^), respectively, while a typical ca. 15 μm-long conidium or ascospore exhibits a projection size of approximately 4600 pixels (approximately 38 μm^2^). One pixel corresponds to approximately 0.0083 μm^2^. The error of the measurement with the current software was evaluated by analysing the given scale bar of 20 µm (right bottom corner); the standard deviation of the measurements gave 0.42 pixels, which is 0.19%.


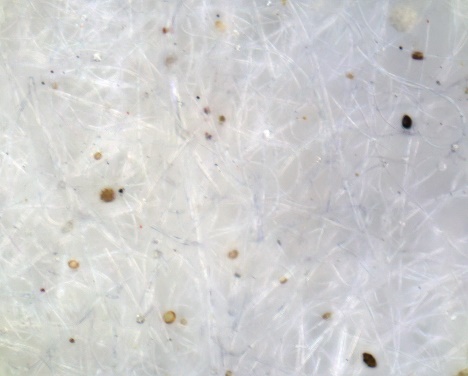


**(a)**







**(c)**

**(b)**

**Supplementary Figure S6** Comparison of the present coloured fungal spore counts and previous spore counts from Igarashi et al. (2019)^13^. (a), (b) and (c) display the original OM image, counting results by the previous analysis in Igarashi et al. (2019)^13^ and counting results of the present analysis, respectively. The previous counting method yields 20 particles, while the present counting method yields 35 particles (in this case, a difference of approximately 1.8 times).

**
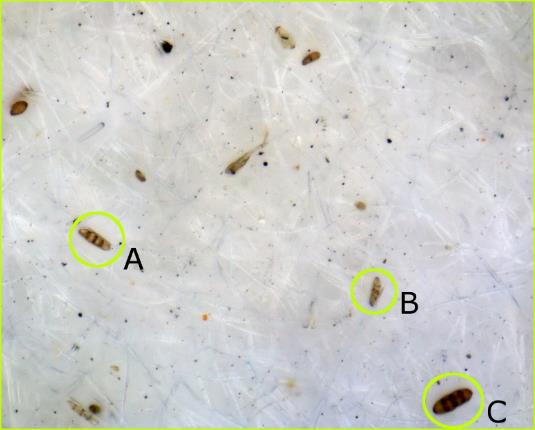
**

**Supplementary Figure S7** Three coarse particles (A, B and C) were analysed for size measurement 10 times. Two spores with lengths of 150.5 pixels (13.7 µm) and 191.4 pixels (17.4 µm) gave areas of 9581±230 pixels (relative error = 2.4%) and 5515±218 pixels (relative error = 3.9%), respectively. The largest spore Particle C exceeded the current measurement scale, which certainly represents a limitation of the current counting methodology. In total, simple summation of the errors in scale measurement and replication produce an error of less than 10%; for Particle A scale error (1.4%) + replication error (2.4%) = 3.8% and for Particle B scale error (3.5%) + replication error (4.0%) = 7.5%, respectively. Particle C exceeded the scale of the present counting method. Therefore, fungal spore counting involves an error of approximately 10%. The data reveal the current limitations of the methodology employed.


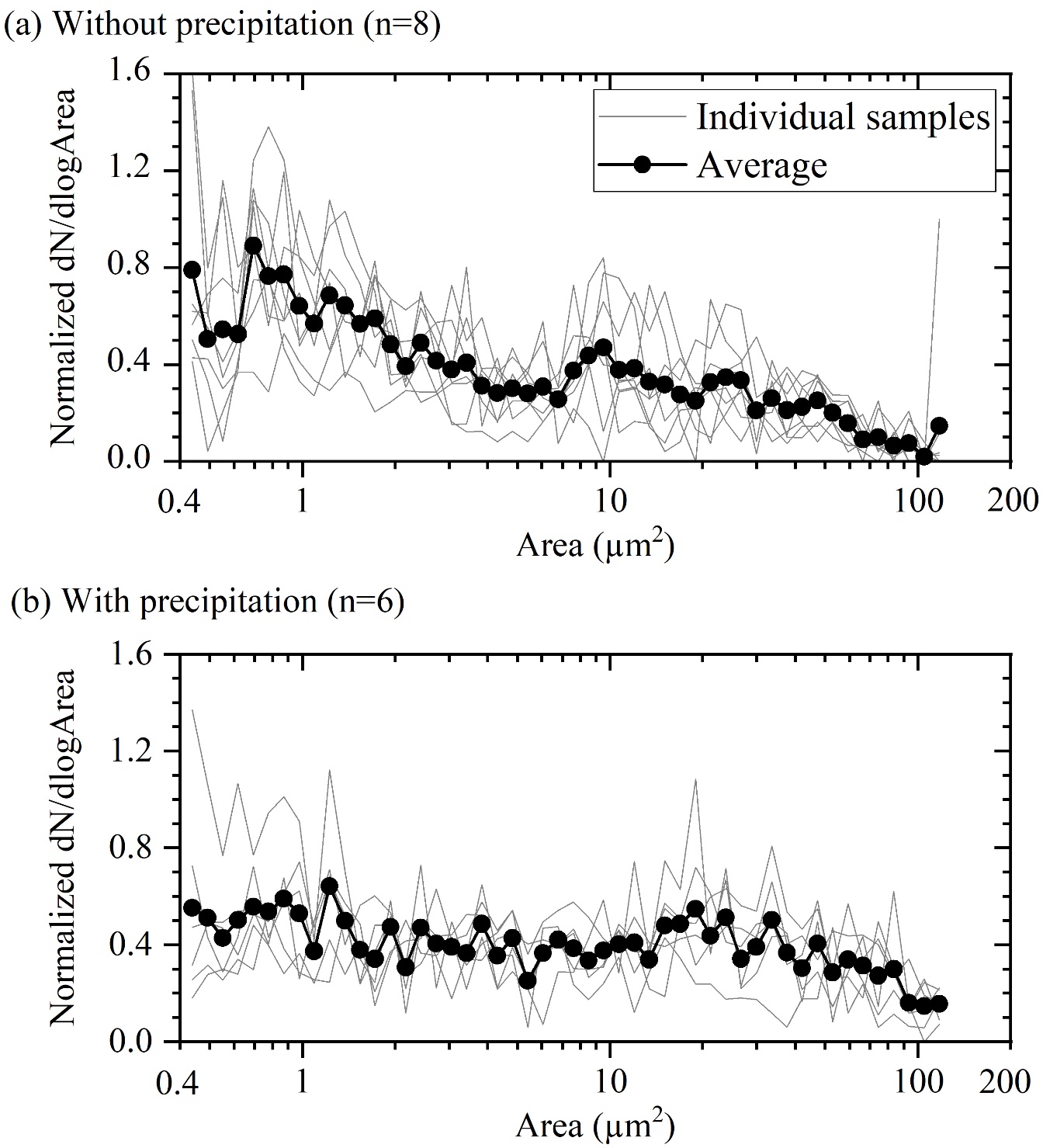


**Supplementary Figure S8** Normalized (divided by total counts) number concentrations (Y) plotted versus spore size (X), giving the size distributions of fungal particles collected on the HV filters (n=6 and 8 rain (bottom) and no rain (top), respectively) obtained in 2016 by image analysis. The bin size of the horizontal axis (X; dlog Area) is 0.05 on the scale of the base 10 logarithm. Analysed optical microscopic images were taken from the same filter samples as those shown in Figure 2. The size of each fungal particle is expressed in terms of the projected area. Particles greater than approximately 120 μm^2^ were cut off to avoid overlapping images of particles. The data were also used to produce Figure 5.


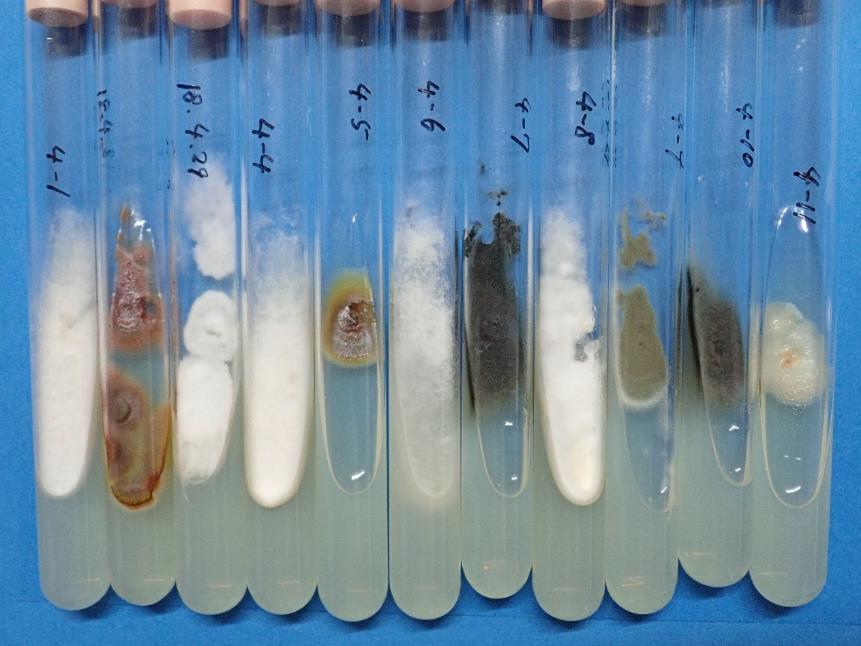


**Supplementary Figure S9** Examples of incubated samples. These filamentous fungi taken from the HV filter samples collected in 2016 were subjected to DNA analyses for species identification. From left: *Pestalotiopsis microspore; Fusicolla* sp.; *Toxicocladosporium irritans; Pestalotiopsis microspore; Fusicolla* sp.; *Arthrinium phaeospermum*; *Xylomelasma* sp.; *Pestalotiopsis microspore; Talaromyces purpureogenus*; *Xylomelasma* sp. and *Fusarium merismoides*.


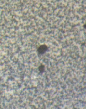
　　
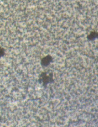
　　
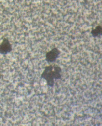
　　
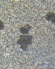


-16°C -18°C -19°C -20°C

**Supplementary Figure S10** An example of a preliminary experiment on ice nucleation of fungal spore (basidiospore) ice growth on a spore of *Psathyrella* *cineraria* collected in the Tsukuba Botanical Garden, Tsukuba, Ibaraki. The fungal spore sample was set in a cooling stage (Linkam Scientific Instrument Ltd: THMS600) incorporated into an optical microscope (Carl Zeiss Axio Imager M2 m), and the temperature and water vapour inside the stage were adjusted by a PC control. The stage temperature was initially set at -8 or -14 °C, and then the stage was cooled gradually at a rate of 5 °C min^-1^, and a video was captured by using software (Bandicam). Microscopic magnification ranging from ×50 to ×200 was chosen according to the sample condition. Judgement of the nucleation onset was performed manually by watching the video record. In this case, we consider that at -18 °C (dew point: -13 °C), ice crystal growth started.

**Supplementary Table S1** Sampling details at the Namie and Kawamata sites: sampling location, weather condition, sampling span, total volume of sampled air and radiocaesium concentrations for the survey of the weather dependence of resuspension from the forest in the summer of 2014. Samples were collected in the contaminated forests in the restricted region of Fukushima Prefecture.

| Location & condition | | Observation span start* | Observation span end* | Sampling length | Air volume | ^137^Cs concentration in air | Error | ^134^Cs concentration in air | Error |
| --- | --- | --- | --- | --- | --- | --- | --- | --- | --- |
|  |  | (YYYY/MM/DD hh:mm) | (YYYY/MM/DD hh:mm) | (h) | (m^3^) | (Bq m^-3^) | (Bq m^-3^) | (Bq m^-3^) | (Bq m^-3^) |
| Namie (Deciduous forest) | Without  Rain | 2014/6/6 13:44 | 2014/6/13 13:44 | 91.5 | 3844 | 8.69×10^-4^ | 9.58×10^-6^ | 3.47×10^-4^ | 4.63×10^-6^ |
|  |  | 2014/6/13 14:12 | 2014/6/20 14:28 | 8.1 | 340.0 | 1.23×10^-3^ | 3.14×10^-5^ | 5.96×10^-4^ | 1.21×10^-6^ |
|  |  | 2014/6/20 14:40 | 2014/6/29 13:59 | 36.7 | 1542 | 1.23×10^-3^ | 1.36×10^-5^ | 4.35×10^-4^ | 2.91×10^-6^ |
|  |  | 2014/6/29 14:22 | 2014/7/4 13:48 | 29.6 | 1242 | 1.08×10^-3^ | 1.49×10^-5^ | 4.28×10^-4^ | 2.27×10^-6^ |
|  |  | 2014/7/4 14:11 | 2014/7/12 14:22 | 49.7 | 2089 | 1.19×10^-3^ | 1.53×10^-5^ | 4.39×10^-4^ | 4.02×10^-6^ |
|  |  | 2014/7/12 14:28 | 2014/7/18 14:07 | 22.3 | 934.7 | 1.67×10^-3^ | 2.66×10^-5^ | 6.27×10^-4^ | 3.67×10^-6^ |
|  | With rain | 2014/6/6 13:39 | 2014/6/13 14:01 | 76.8 | 3226 | 6.46×10^-4^ | 9.17×10^-6^ | 2.42×10^-4^ | 1.89×10^-6^ |
|  |  | 2014/6/13 14:22 | 2014/6/20 14:35 | 160.0 | 6720 | 2.82×10^-4^ | 4.61×10^-6^ | 1.02×10^-4^ | 6.93×10^-7^ |
|  |  | 2014/6/20 14:39 | 2014/6/29 14:14 | 178.6 | 7500 | 3.80×10^-4^ | 3.70×10^-6^ | 1.36×10^-4^ | 1.39×10^-6^ |
|  |  | 2014/6/29 14:21 | 2014/7/4 14:00 | 89.8 | 3772 | 6.98×10^-4^ | 6.33×10^-6^ | 2.61×10^-4^ | 2.57×10^-6^ |
|  |  | 2014/7/4 14:08 | 2014/7/12 14:07 | 142.2 | 5972 | 6.66×10^-4^ | 6.65×10^-6^ | 2.42×10^-4^ | 3.51×10^-6^ |
|  |  | 2014/7/12 14:30 | 2014/7/18 14:13 | 121.4 | 5097 | 3.29×10^-4^ | 5.38×10^-6^ | 1.28×10^-4^ | 8.34×10^-7^ |
| Kawamata (Coniferous forest) | Without  Rain | 2014/6/6 11:24 | 2014/6/13 12:20 | 82.8 | 3480 | 1.10×10^-4^ | 4.18×10^-6^ | 3.75×10^-5^ | 4.31×10^-6^ |
|  |  | 2014/6/13 12:25 | 2014/6/20 10:36 | 12.3 | 516.9 | 2.48×10^-4^ | 2.04×10^-5^ | 9.87×10^-5^ | 1.93×10^-5^ |
|  |  | 2014/6/20 10:45 | 2014/6/29 9:25 | 31.1 | 1306 | 1.46×10^-4^ | 9.88×10^-6^ | 6.45×10^-5^ | 1.04×10^-5^ |
|  |  | 2014/6/29 10:02 | 2014/7/4 10:30 | 30.8 | 1292 | 1.25×10^-4^ | 8.68×10^-6^ | 4.54×10^-5^ | 9.36×10^-6^ |
|  |  | 2014/7/4 10:37 | 2014/7/12 9:59 | 47.5 | 1994 | 1.71×10^-4^ | 7.22×10^-6^ | 7.34×10^-5^ | 7.59×10^-6^ |
|  |  | 2014/7/12 10:10 | 2014/7/18 10:19 | 25.3 | 1062 | 2.58×10^-4^ | 1.54×10^-5^ | 9.46×10^-5^ | 1.30×10^-5^ |
|  |  | 2014/7/18 10:24 | 2014/8/1 10:00 | 36.1 | 1518 | 3.68×10^-4^ | 9.41×10^-6^ | 1.18×10^-4^ | 6.91×10^-6^ |
|  | With  Rain | 2014/6/6 11:15 | 2014/6/13 12:11 | 71.2 | 2992 | 1.17×10^-4^ | 4.91×10^-6^ | 4.31×10^-5^ | 4.74×10^-6^ |
|  |  | 2014/6/13 12:25 | 2014/6/20 10:27 | 125.4 | 5267 | 1.33×10^-4^ | 4.20×10^-6^ | 3.86×10^-5^ | 3.09×10^-6^ |
|  |  | 2014/6/20 10:44 | 2014/6/29 9:13 | 150.6 | 6324 | 1.28×10^-4^ | 2.62×10^-6^ | 5.10×10^-5^ | 2.19×10^-6^ |
|  |  | 2014/6/29 9:41 | 2014/7/4 10:15 | 74.2 | 3115 | 2.34×10^-4^ | 5.42×10^-6^ | 8.39×10^-5^ | 5.09×10^-6^ |
|  |  | 2014/7/4 10:35 | 2014/7/12 9:49 | 118.2 | 4965 | 1.62×10^-4^ | 4.67×10^-6^ | 4.61×10^-5^ | 3.93×10^-6^ |
|  |  | 2014/7/12 10:11 | 2014/7/18 10:10 | 97.5 | 4097 | 1.43×10^-4^ | 5.45×10^-6^ | 5.70×10^-5^ | 4.72×10^-6^ |
|  |  | 2014/7/18 10:26 | 2014/8/1 9:53 | 245.7 | 10321 | 1.22×10^-4^ | 2.02×10^-6^ | 4.40×10^-5^ | 1.69×10^-6^ |

* The real sampling duration was controlled by the rain sensor

**Supplementary Table S2** Sampling details at the Namie site (forest and bare ground): weather conditions, sampling span, total volume of sampled air and radiocaesium concentrations for the survey of the weather dependence of resuspension from the forest in the summer and autumn of 2016. Samples were collected in the contaminated forests and the school ground in the restricted region of Fukushima Prefecture.

| Weather condition |  | Observation span start* | Observation span end* | Day or Night | Sampling length | Air volume | ^137^Cs activity concentration  in air | Error | Coloured particle concentration | Error | Median size of particle |
| --- | --- | --- | --- | --- | --- | --- | --- | --- | --- | --- | --- |
|  |  | (YYYY/MM/DD hh:mm) | (YYYY/MM/DD hh:mm) |  | (h) | (m^3^) | (Bq m^-3^) | (Bq m^-3^) | (counts m^-3^) | (counts m^-3^) | (µm^2^) |
| Without rain | G | 2016/8/24 6:20 | 2016/8/25 18:20 | Day | 24.0 | 1296 | 1.79×10^-4^ | 1.25×10^-5^ | 3.39×10^4^ | 1.69×10^4^ | 2.73 |
|  | G | 2016/8/31 18:19 | 2016/9/2 6:19 | Night | 24.0 | 1296 | 7.15×10^-4^ | 1.54×10^-5^ | 1.57×10^5^ | 2.62×10^4^ | 2.17 |
|  | G | 2016/9/4 18:16 | 2016/9/6 6:16 | Night | 24.0 | 1296 | 5.00×10^-4^ | 1.46×10^-5^ | 1.12×10^5^ | 2.00×10^4^ | 3.83 |
|  | G | 2016/9/5 6:21 | 2016/9/6 18:21 | Day | 24.0 | 1296 | 1.10×10^-4^ | 1.08×10^-5^ | 8.52×10^4^ | 1.93×10^4^ | 1.72 |
|  | G | 2016/10/1 6:21 | 2016/10/2 18:21 | Day | 24.0 | 1296 | 4.47×10^-4^ | 1.10×10^-5^ | 8.11×10^4^ | 1.42×10^4^ | 2.17 |
|  | G | 2016/9/24 18:18 | 2016/9/26 6:18 | Night | 24.0 | 1296 | 1.12×10^-6^ | 1.52×10^-5^ | 7.31×10^4^ | 1.22×10^4^ | 5.39 |
|  | G | 2016/10/9 18:07 | 2016/10/13 6:07 | Night | 48.0 | 2016 | 3.70×10^-4^ | 6.29×10^-5^ | 4.52×10^4^ | 1.12×10^4^ | 1.38 |
|  | G | 2016/10/10 6:17 | 2016/10/11 18:16 | Day | 24.0 | 1007 | 3.40×10^-4^ | 1.05×10^-4^ | 4.97×10^4^ | 6.58×10^3^ | 1.38 |
| With rain | F | 2016/8/11 13:33 | 2016/9/2 12:41 | Both | 161.5 | 6781 | 7.75×10^-4^ | 5.23×10^-6^ | 2.69×10^4^ | 1.78×10^3^ | 4.29 |
|  | F | 2016/9/2 12:46 | 2016/10/5 11:22 | Both | 204.0 | 8569 | 1.13×10^-3^ | 6.80×10^-6^ | 2.01×10^4^ | 2.03×10^3^ | 4.81 |
|  | F | 2016/10/5 11:33 | 2016/10/29 11:35 | Both | 27.3 | 1146 | 5.88×10^-4^ | 1.73×10^-5^ | 4.41×10^4^ | 8.15×10^3^ | 1.37 |
|  | G | 2016/8/11 14:33 | 2016/9/2 14:25 | Both | 167.5 | 7034 | 1.83×10^-4^ | 2.66×10^-6^ | 1.15×10^4^ | 2.04×10^3^ | 6.77 |
|  | G | 2016/9/2 14:29 | 2016/10/5 13:39 | Both | 216.5 | 9094 | 2.60×10^-4^ | 3.34×10^-6^ | 1.45×10^4^ | 2.96×10^3^ | 8.51 |
|  | G | 2016/10/5 13:44 | 2016/10/29 12:55 | Both | 33.3 | 1397 | 3.25×10^-4^ | 1.20×10^-5^ | 4.41×10^4^ | 8.79×10^3^ | 6.77 |

* The real sampling duration was controlled by the rain sensor
